# A Wireless, Battery‐Free Artificial Throat Patch with Deep Learning for Emotional Speech Recognition

**DOI:** 10.1002/advs.202516617

**Published:** 2026-01-28

**Authors:** Bingxin Xu, Guanming Lin, Xiaohui Hong, Chuting Liu, Xinyi Qu, Kangjian Jiao, Lei Zhang, Bingzhe Xu, Jun Yan, Yancong Qiao, Xudong Lin

**Affiliations:** ^1^ Guangdong Provincial Key Laboratory of Sensor Technology and Biomedical Instrument School of Biomedical Engineering Shenzhen Campus of Sun Yat‐Sen University Shenzhen China; ^2^ The Second Clinical Medical College Guangzhou University of Chinese Medicine Guangzhou China

**Keywords:** artificial throat, bioelectronics, deep learning, emotion recoginition, speech recoginition

## Abstract

Technologies for sensing and translating throat signals into speech have advanced the communication for people with vocal impairments. However, the practical deployment of these technologies is currently hindered by tethered system, bulky components and the lack of emotional recognition capability. Here, we demonstrate a wireless, battery‐free artificial throat patch system (ATPS) coupling with deep learning method to enable simultaneous recognition of speech and emotion. The sensing module of the ATPS integrates a carbon nanotube‐based thin‐film strain sensor and a miniaturized flexible printed circuit board (FPCB). Real‐time sensing and transmission of throat signals is facilitated by a smartphone‐linked system incorporating an FPCB with an embedded near‐field communication antenna and low‐power electronic components. Furthermore, the information‐rich sensor readouts are further extracted and successfully demonstrated to be sufficient to emotional recognition of speech by implementing a hybrid deep learning architecture. This proposed system expands the arsenal of tools available for mute patients in speech recognition and communication and further realizes personalized clinical implementations.

## Graphical abstract

In this work, Xu and co‐workers develop a wireless, battery‐free artificial throat patch system (ATPS) consisting of a carbon nanotube‐based thin‐film strain sensor and a miniaturized flexible printed circuit board, to enable real‐time sensing of throat signals. Coupling with a deep learning method, this system is successfully demonstrated for simultaneous recognition of speech and emotion.

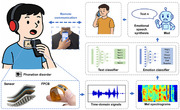

## 1 Introduction

Emotion and language are fundamental to human social interaction, with emotional expression accounting for approximately 38% of interpersonal communication [1, 2, 3, 4]. It is worth noting that patients with vocal impairments after injuries or disorders may suffer heavily from both physical and mental burdens. Though various technologies have been proposed to improve the quality of communications among these patients, recent studies have shown that prolonged emotional expression disorders not only exacerbate anxiety but also significantly accelerate pathological progression in chronic conditions, such as cardiovascular diseases [5, 6]. Therefore, addressing this dual challenge in individuals with vocal impairments remains a critical unmet need.

Recent progress in sensing systems has enabled simple communications for mute individuals [7]. For example, implanted systems based on electrophysiological recording technologies coupled with advanced decoding algorithms have been proposed for speech recognition [8, 9, 10, 11]. Specifically, several silent speech recognizers have been developed based on monitoring the human cortical activities in language‐associated brain regions [8, 9]. However, the widespread adoption of these systems remains limited by the invasiveness and inherent complexity. Although numerous non‐invasive approaches, such as lip‐reading strategy or eye‐tracking technology, have also been developed to enable silent communication, the prohibitive costs and the lack of portability in fact limit their translational implementation [12, 13]. Recently, there have been great efforts for developing wearable artificial throat systems based on throat signals, which contain throat vibrations, breathing frequency changes, and speech rate fluctuations, aiming to effectively reconstruct the vocal function of speech‐impaired people in a continuous and comfortable way [14, 15]. Based on the capacitive, piezoelectric, electromagnetic, and piezoresistive materials and specific sensing structures, lightweight sensors have been proposed in these systems for monitoring throat signals with high signal‐to‐noise ratio [16, 17, 18, 19, 20, 21]. Moreover, machine learning or deep learning models have also been employed to achieve high accuracy and speed in large word‐sets recognition [22]. Even though these strategies have greatly advanced the electrolarynx in the reconstruction of voice information, the level of system integration remains insufficient. Most proposed platforms are tethered and battery powered, which heavily limit patients’ mobility, induces stress, causes mechanical damage to the device, and require advanced cable management [18, 19, 20, 21]. These limitations essentially eliminate the free movements of the patients and the further practical applications of these systems. Therefore, the development of a tether‐free, battery‐free, and fully integrated electronic patch capable of continuously monitoring throat signals and recognizing speech using advanced algorithms is an unmet need and has not been fully realized.

With the rising global focus on mental health, there is a growing need for accurate emotion recognition in applications such as disease treatment, immersive daily interaction services, and remote communication, especially for individuals with speech impairments [1, 23]. However, the inherent abstraction, complexity, and highly personalized nature of emotions present significant challenges for emotion recognition in this population [24]. Current approaches have primarily focused on analyzing single or multimodal data, such as speech, facial expressions, and brain activities [25, 26, 27, 28]. In particular, the advances in multimodal sensors and hybrid electronics have facilitated emotion recognition with high accuracy [29, 30, 31, 32, 33, 34, 35]. While powerful, these technologies are often limited by system complexity, wearable inconvenience, and high costs, which also hinder widespread adoption [36, 37, 38, 39, 40]. Hence, a simple, low‐cost, integrated wearable device based on laryngeal vibration for accurate emotional speech recognition in patients remains attractive and is also yet to be developed.

To fill this gap, we propose a wireless, battery‐free artificial throat patch system (ATPS) to emotionally recognize the speech of individuals with voice defects. The core of the ATPS is an integrated patch containing a thin‐film strain sensor and a miniaturized flexible printed circuit board. To enable sensing the highly weak throat signals, carbon nanotube‐Polydimethylsiloxane composite based thin films with a micropillar array structure is employed to develop the strain sensor. Additionally, an ethyl cellulose ‐coated multi‐walled carbon nanotube layer is introduced to optimize the interface matching and sensing. Wirelessly monitoring of sensor readouts and communicating with the smartphone are achieved through the FPCB, which incorporates low‐power electronics with transmitting circuit modalities. Furthermore, the Mel Frequency Cepstral Coefficients of the throat signals are extracted and classified using a hybrid deep learning model based on the classifier (FC Layer + Softmax) to identify both text and emotional content. In this proof‐of‐concept study, we validate the functionality and demonstrate the applications of the ATPS for emotionally restoring the speech. We envision that our ATPS device will expand the toolkit available for laryngeal sensing and further facilitate tailored application schemes to improve life quality for disabled populations.

## 2 Results

### 2.1 System Design of the ATPS

**FIGURE 1:**
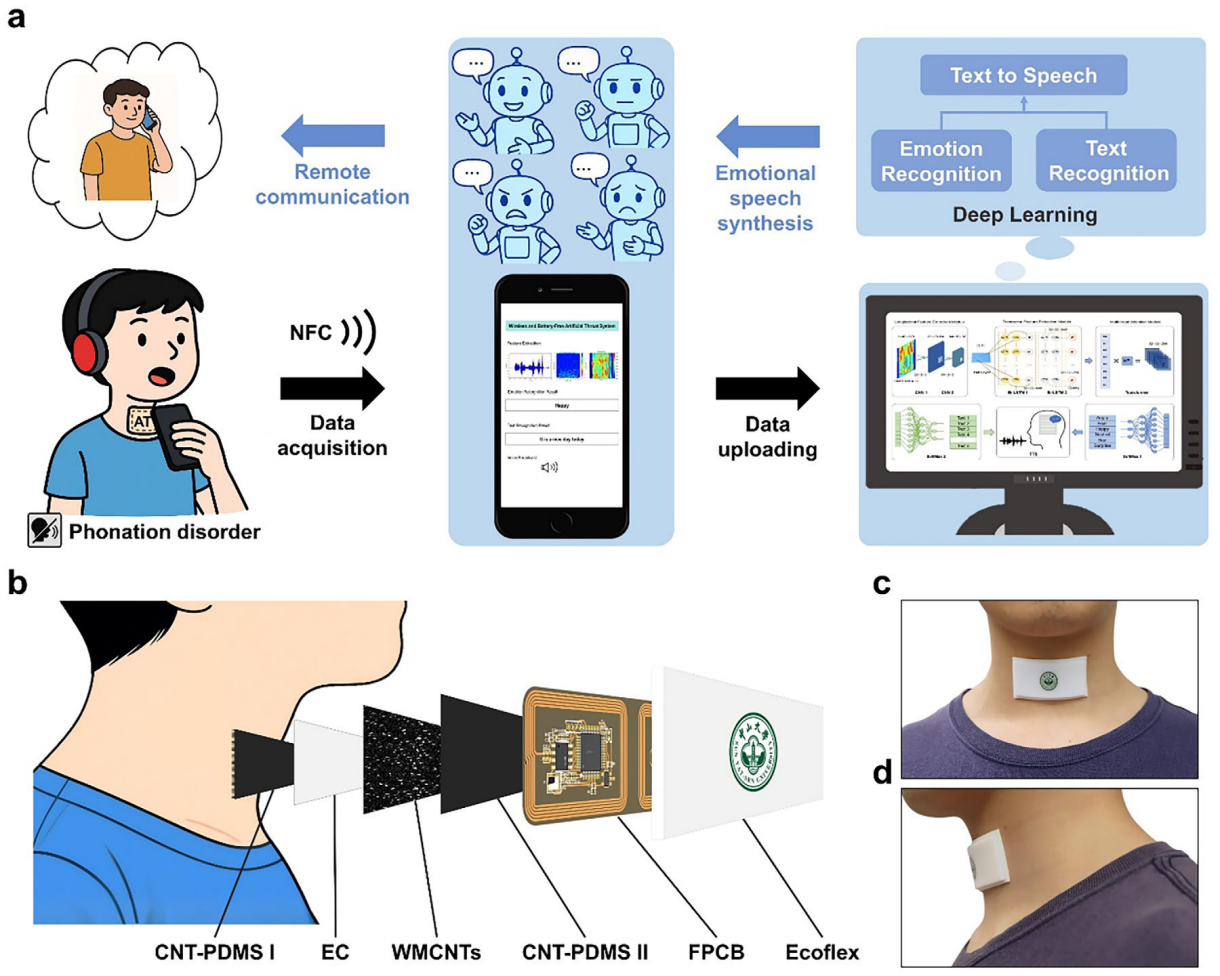
System design of the ATPS. a) Schematic of the artificial throat patch system (ATPS) with deep learning for emotional speech recognition. b) Exploded view illustration of the ATPS, including CNT‐PDMS I (plane film), ethyl cellulose (EC) electrospum nanofiber, multi‐wall carbon nanotubes (MWCNTs), CNT‐PDMS II (with microstructure), FPCB and Ecoflex. c‐d) Photographs showing the ATPS firmly adhered to the throat.

To enable wireless, battery‐free monitoring and emotionally recognizing the speech of individuals with vocal impairments to express emotional language, we developed an artificial throat patch system (ATPS) (Figure 1a). It contained three functional modules, including a sensing module, a miniaturized flexible printed circuit board (FPCB) with a near‐field communication (NFC) terminal, and a smartphone worked with a deep learning algorithm. As the core component of the ATPS, the strain sensor hybridized multiple layers of flexible structural substrates, integrating carbon nanotube (CNT)‐Polydimethylsiloxane (PDMS) composite based functional thin films and an ethyl cellulose (EC)‐coated multi‐walled carbon nanotubes (MWCNTs) layer to realize the sensitive detection of tiny vibrations from the throat as shown in Figure 1b. In the ATPS, the fluctuations of the sensor resistance could be easily read by the miniaturized FPCB with NFC technology. In addition, a hybrid deep learning model was also developed here to identify both text and emotional content of the silent speech based on the readouts from the sensor. The final synthesized emotional speech would be generated further via using Text‐to‐Speech (TTS) technology. Moreover, due to the light‐weighting (approximately 0.8 g), thin profile of the FPCB (approximately 200 µm) and the fully flexible, battery‐free design of the sensing layers, our patch could be comfortably attached to the user's throat (Figure 1c,d).

### 2.2 Sensor Design and Characterization

**FIGURE 2:**
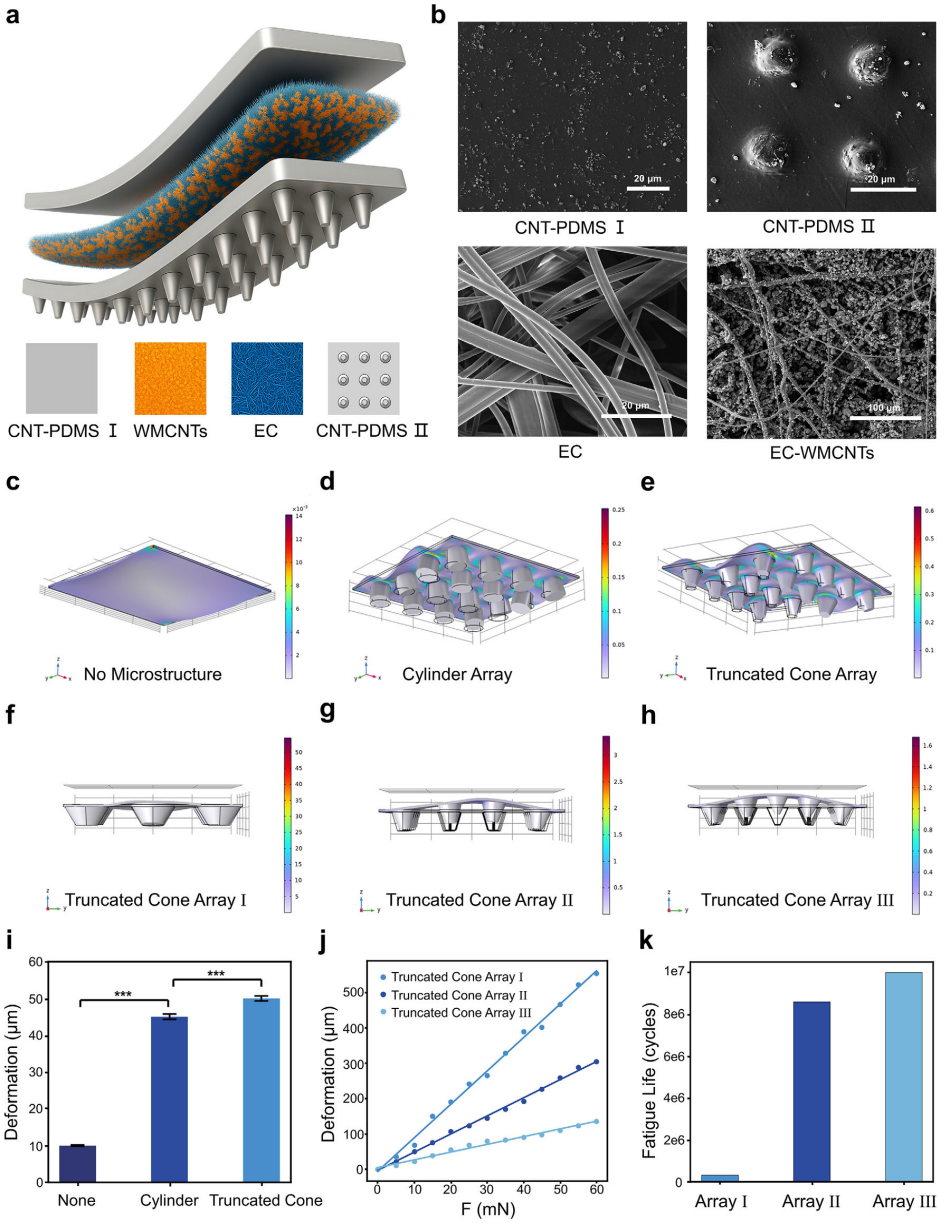
Design and simulation of sensors. a) Schematic diagram of the mechanical sensor with a sandwich architecture. b) SEM images of CNT‐PDMS plane film (CNT‐PDMS I), CNT‐PDMS with microstructure (CNT‐PDMS II), electrospun ethyl cellulose (EC) nanofiber and electrospun EC nanofiber uniformly coated with MWCNTs. c‐e) Finite element analysis (FEA) of strain distribution in CNT‐PDMS films with (c) flat surfaces, (d) cylindrical microarrays, and (e) inverted truncated cone microarrays under mechanical loading. f–h) FEA of CNT‐PDMS films with inverted truncated cone microarrays of varying sizes. i) Surface displacement profiles of CNT‐PDMS films with different surface microstructures under micro‐load conditions. j) Load‐dependent deformation of CNT‐PDMS films featuring inverted truncated cone arrays with different dimensions. k) Simulated cyclic stability of CNT‐PDMS films of dimensionally varied inverted truncated cone microarrays. Kruskal‐Wallis H test *n* = 10, collected from 10 devices; ^***^ *p *< 0.001; error bars indicated standard deviation.

To enable efficient mechanical sensing on the irregular surfaces of the human throat, a flexible strain sensor was designed and fabricated in our system. Figure 2a showed the multi‐layered structure of the sensor based on MWCNTs (8‐15 nm diameter, 30–50 µm length), assembled in a sandwich configuration with a CNT‐ PDMS composite plane film in the top layer, an electrospun EC fiber film coated with MWCNTs in the middle layer, and a CNT‐PDMS composite film with specifically designed microstructures in the bottom layer. A simple method was employed here for the rapid and low‐cost manufacturing of the strain sensors with multilayer functional structures. Each thin film layer was prepared through a series of straightforward coating processes and was further characterized using scanning electron microscopy (Figure 2b). The CNT‐PDMS composite layer served as a conductive substrate, exhibiting a smooth surface morphology with corrugated and elongated multi‐walled carbon nanotubes (MWCNTs) (Figure S1). These MWCNTs were distributed either on the PDMS surface or embedded within the matrix, adopting winding, interwoven, or stacked configurations, while their termini protruded from the PDMS interface as shown in Figure 2b. The electrospun EC membrane exhibited a continuous and homogeneous nanofibrous architecture with randomly interwoven 3D networks, demonstrating high porosity, loosely packed structures, ultrasmooth fiber surfaces, and uniform spatial distribution. This unique architecture, resembling non‐woven textiles, facilitated conformal contact with various substrates, making it particularly suitable as an interfacial buffer layer. Through optimized spray‐coating, MWCNTs were precisely deposited onto the electrospun fibers to offer its electrical conductivity. SEM analysis revealed that the fibers self‐assembled into a randomly cross‐linked 3D network architecture. And the MWCNTs were uniformly coated at fiber surfaces and nodal intersections. The cross‐sectional SEM images further visualized the internal morphology of different layers of the sensing structure in our system (Figure S2). This hierarchical distribution established percolative conductive pathways, which accounted for the observed enhancement in electromechanical sensing performance [41, 42, 43].

Dystonia and functional aphonia account for nearly half of all voice disorders [44, 45]. When attempting to speak, the vocal fold vibration frequency of these patients is similar to that of normal individuals, but their naturally small amplitude may be reduced further [46, 47, 48]. Therefore, a CNT‐PDMS composite film containing micropillars was designed in our system for allowing effectively monitoring such tiny deflection, which was further validated with various simulation and experimental tests (Figure 2c–k). These microstructures were fabricated via simple molding (Figure S3). As shown in Figure S4, super‐depth scanning micrographs revealed an array of arranged and truncated cone shapes of the micropillars with a height of ∼80 µm. A 3D finite element analysis was carried out to investigate the mechanical deformation of the micropillar arrays in the CNT‐PDMS II layer (Figure 2c–h; Figure S5). To simulate laryngeal vibrations, a dynamic load of 10sin (400πt) mN superimposed on a static preload ranging from 0 to 60 mN was applied, enabling comparative analysis of the mechanical responses of thin films with different microstructures (Tables S1 and S2). We examined the vertical reflections as a function of different structures and acting forces. The results showed that the truncated cone structure exhibited higher stress concentration at its narrower base region compared to cylindrical structures, resulting in more pronounced localized deformation (Figure 2i). The maximum displacement of the CNT‐PDMS II layer was also adjusted by varying the applied forces and truncated cone sizes (Figure 2j). Of note, further finite‐cycle fatigue simulations under 50 mN loading revealed that stress concentration effects lead to a positive correlation between the truncated cone's base diameter and the fatigue life (Figure 2k). A deformation test of the sensing film was then performed in this study. As shown in Figure S6, the membrane deformation was adjusted by varying the applied vertical force. And it was noted that the experimental results showed substantial consistency with the simulation results. It provided strong evidence for the reliability of our simulation‐based parameter optimization strategy. Consequently, a truncated cone structure with intermediate dimensions was used in the ATPS based on a comprehensive evaluation of both sensitivity and durability.

**FIGURE 3:**
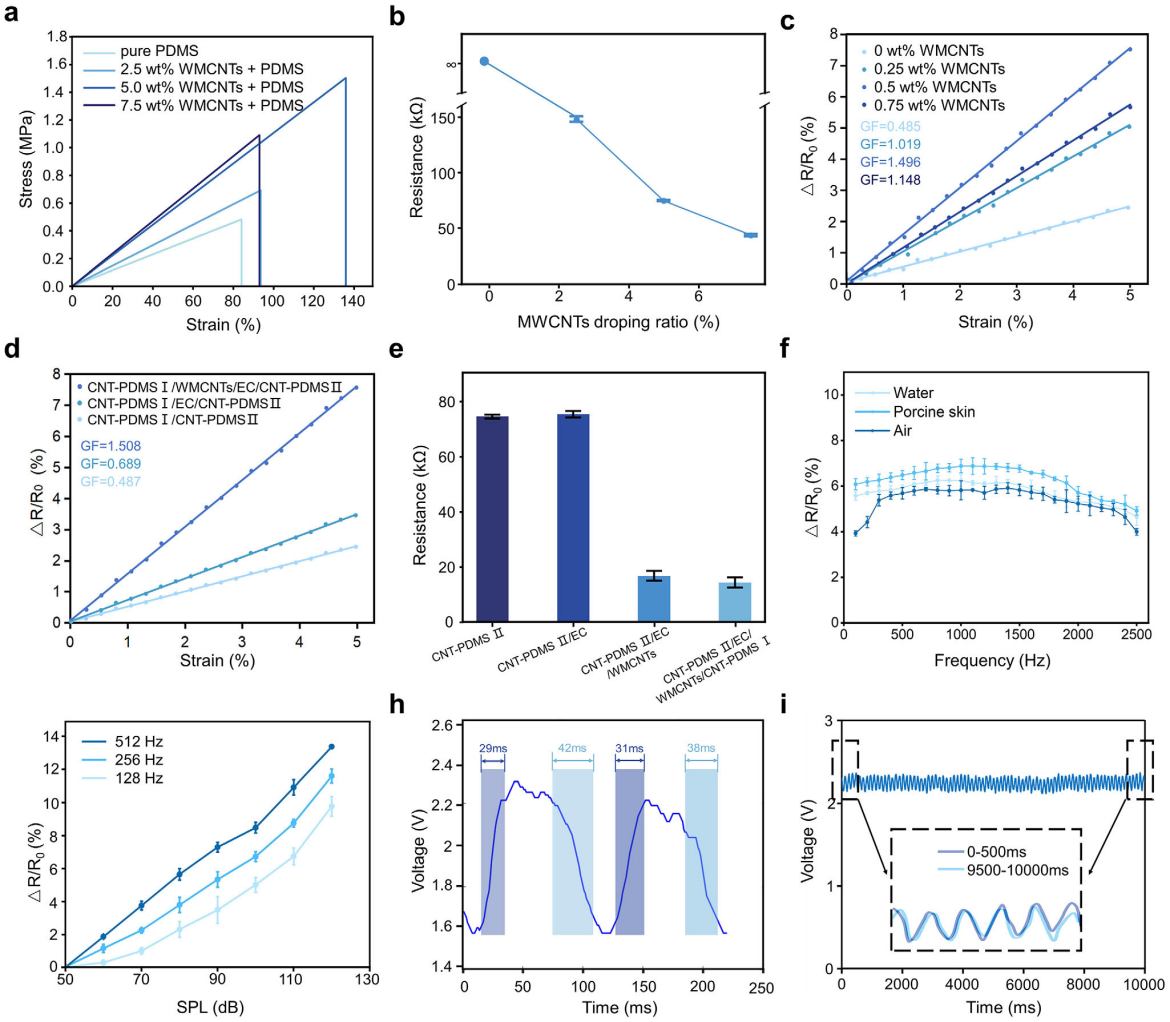
Characterization of sensors. a) Stress–strain curves of CNT‐PDMS films with varying MWCNTs doping levels (0, 2.5, 5, 7.5 wt.%) under uniaxial tensile loading. b) Statics resistance of CNT‐PDMS films as a function of MWCNTs concentration (*n* = 5). c) The strain response of EC under different doping concentrations of WMCNTs (0, 0.25, 0.5, 0.75 wt.%). d) Micro‐strain responses of devices with different assembly strategies. e) Static resistances of devices with different assembly strategies (*n* = 5). f) The frequency response of the final sensor that covers the range of human voice frequencies (90 dB, 100–2500 Hz) in different transmitting media. g) Intensity response of the final sensor at three different typical frequencies (128, 256, and 512 Hz). h) Response and recovery curve of the sensor under a sound stimulus (90 dB, 200 Hz). i) The response curve of the sensor under constant excitation (90 dB, 256 Hz), showing high response consistency. *n* = 10, collected from ten devices; error bars indicated standard deviation.

To fully validate the functionality of the sensor patch, a series of mechanical and electrical tests were conducted. As shown in Figure 3a, MWCNTs doping significantly improved the mechanical properties of PDMS. With increasing doping rates, the tensile strength of PDMS increased from 0.48 to 1.65 MPa, the fracture strain from 85% to 137%, and toughness from 0.21 to 1.03 MJ/m^3^ (Table S3). The optimal doping rate of MWCNTs was identified to be 5 wt.% regarding its electrical and mechanical performance (Figure 3b). We then tested the sensing performance of the EC electrospun fiber membrane uniformly coated with MWCNTs powder (Figure 3c). The results demonstrated that the fiber layer's resistance exhibited a linear response within the 0–5% strain range. And increasing the dopant concentration enhanced the conductive network density, resulting in improved sensitivity (Gauge Factor enhanced from 0.485 to 1.496). However, at 0.75 wt.% doping rate, a sensitivity reduction occurred due to conductive path oversaturation. This phenomenon might result from MWCNTs aggregation on the electrospun EC fiber surface, leading to either localized “short‐circuiting” or uneven conductive path distribution, which ultimately diminished resistance change sensitivity under small deformations [49, 50, 51, 52]. Sensors with different assembly schemes were also characterized (Figure 3d). It showed that the introduction of microstructures significantly enhanced the sensitivity of our device, further with the EC/MWCNTs composite layer yielding a 3‐fold improvement. Moreover, electrical characterization revealed that our sandwich‐structured design exhibited reduced static resistance, which allowed a highly stable and sensitive monitoring of the weak signals (Figure 3e).

After ensuring that our patch device was suitable for detection of weak signals, we tested whether the MWCNTs based platform could response to the human throat vibrations with a long ranging frequency. The device was placed 0.5 cm from a loudspeaker to mimic the stimulation with an intensity and frequency comparable to human throat vibrations using a commercial acoustic testing platform (Figure S7 and Note S1). It showed that our device exhibited a significant and stable response to acoustic signals up to 2500 Hz, which generally covers the frequency range of human speech (Figure 3f,g). Standard sensitivity tests in different environment were conducted in this study to further reveal the frequency responses of our sensor (Figure 3f; Figure S6). The results showed that in an environment more closely resembling human tissue (liquid media or porcine skin), the device exhibited higher sensitivity and demonstrated significantly improved stability across a wider frequency range. To further directly demonstrate the strain sensing ability of our system, a strain sensing test at different frequencies was also conducted in this study. The results showed that 90 dB sound pressure have induced a 3.40 ± 0.28% average strain up to 2000 Hz (Figure S8). Moreover, a 30 ± 1.5 ms response time and a 40 ± 2.0 ms recovery time were detected via connecting to a voltage divider circuit with a 3.3 V DC power supply, indicating the high time resolution of the device (Figure 3h). Given that linguistically meaningful units (e.g., phonemes typically last 50–200 ms and syllables often exceed 200 ms), such a response time enables multiple measurements within a single speech unit and accurate tracking of its dynamic acoustic profile [53, 54]. And the sensing consistency was also confirmed under constant stimulation. 2000 cyclic tests revealed that there was no detectable decline in mechanical performance, demonstrating the strong stability of our sensor in the ATPS (Figure 3i; Figure S9).

### 2.3 Circuit Design of FPCB

**FIGURE 4:**
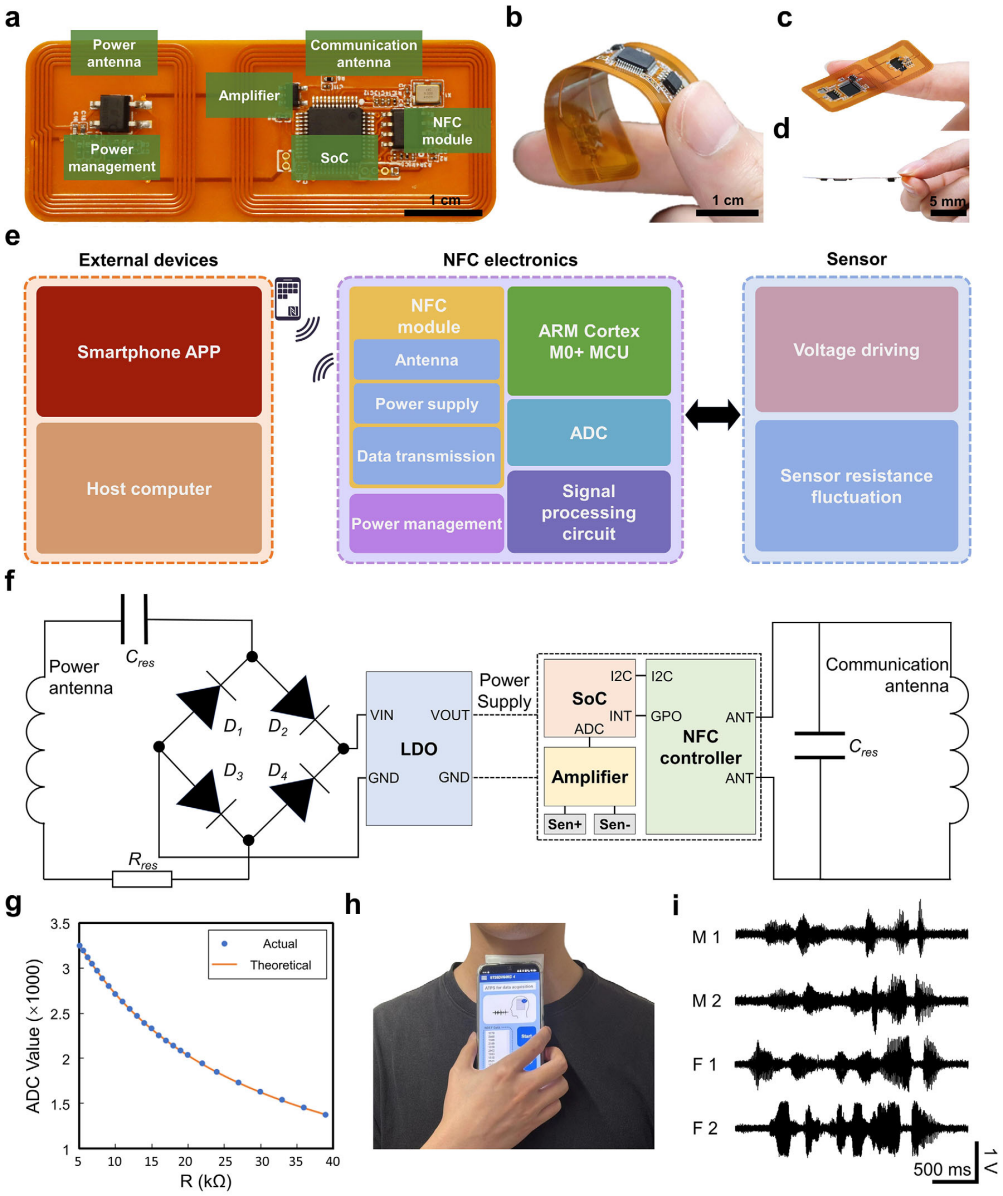
Circuit design of FPCB. a) Photograph of FPCB, showing the overall circuit design and the layout of components, including SoC, NFC module, amplifier and power management. b–d) Photographs showing flexibility (b), miniaturized size (c), and thin board layout (d) of FPCB. e) Block diagram showing key components of the ATPS, including host system, near‐field‐communication low‐power electronics, and sensor. f) Design of the power supply and communication in FPCB. g) Calibration curve of ADC value under known resistance. h) Photograph of a user wears and operates the ATPS. i) The typical throat signals collected through the ATPS from 4 individuals (male 1: 23 years old; male 2: 54 years old; female 1: 21 years old; female 2: 57 years old).

Figure 4a showed the overall view of the FPCB in the ATPS. The FPCB was made from polyimide (PI) with patterned copper traces, including components such as the SoC, amplifiers, and NFC controller (Figure S10). While these components were rigid, the flexible design of the substrate and antennas allowed the miniaturized FPCB to comfortably fit onto the throat (Figure 4b–d). The main electronic system of the FPCB included: 1) A signal acquisition circuit connected to the sensor (resistance‐to‐voltage conversion); 2) A microcontroller (STM32L051, STMicroelectronics) for sampling and compressing the voltage signals from the acquisition circuit; 3) Two NFC antennas, which were directly patterned into the FPCB and tuned to the NFC frequency band (∼13.56 MHz) for power supply and communication; 4) An NFC controller (ST25DV64K), which stored signals received from the microcontroller and transmitted the data via the communication antenna to the NFC terminal; 5) A power management module with a rectifier bridge and low dropout regulator (LDO), which received NFC energy through the power coil and converted it into direction current (DC) voltage (Figure 4e). Compared to Bluetooth and other strategies commonly used in biotechnologies, the NFC design in the ATPS offered the advantages of battery‐free, lightweight operation and high data security [55, 56, 57]. During operation, the FPCB power coil was connected to a fast‐recovery rectifier bridge for high‐frequency rectification, followed by standard voltage regulation to supply power. Sensor data was stored in the NFC module and then transmitted externally via the communication coil (Figure 4f; Figure S11). Circuit performance tests demonstrated high accuracy in both measured values and curve fitting, confirming the FPCB's precise resistance monitoring capability (Figure 4g). A 100% accuracy of ADC reading was maintained at a separation distance of 4 cm from our device (Figure S12). Even under substantial bending, a 100% accuracy of ADC reading would also be maintained up to a separation distance of 3 cm. Moreover. since our sensor was not fully adhered to the FPCB (only fixed and packaged along the two side edges of the sensor), its sensitivity response showed no significant difference compared to that of the bare sensor (Figure S13). As shown in Figure 4h, the integrated ATPS was designed to be attached to the throat with bending deformation. Hence, we further tested the device's output performance during bending deformation. The bending radii (R) corresponding to typical neck curvatures of adult males, adult females, and children (R = 6.35 cm, 5.60 cm, and 3.45 cm, respectively) were employed to bend our device. As shown in Figure S14, there was a slight variation in sensitivity, frequency response, and NFC communication distance of the sensor with different Bending radii (R). Overall, the degree of bending had no significant impact on the sensor's outputs, though it did moderately affect the NFC communication range. However, even under substantial bending (R = 3.45 cm), the NFC communication distance remained up to 3 cm, which would not substantially hinder its daily use. It was noted that our device also showed stable sensing performance in different testing environment (Figure S15). The integration of the FPCB and sensor in the ATPS eventually enabled a straightforward approach to track information‐rich throat signals (Figure 4i). Furthermore, the long‐term stability of the ATPS was also evaluated. As shown in Figure S16, weeks‐long sensing of small sound pressures was enabled by our devices. As a result, our ATPS system exhibited superior performance in the perspective of sensitivity, linearity, response time, response range, and cost (Table S4).

### 2.4 Deep Learning Based Emotional Speech Recognition

**FIGURE 5:**
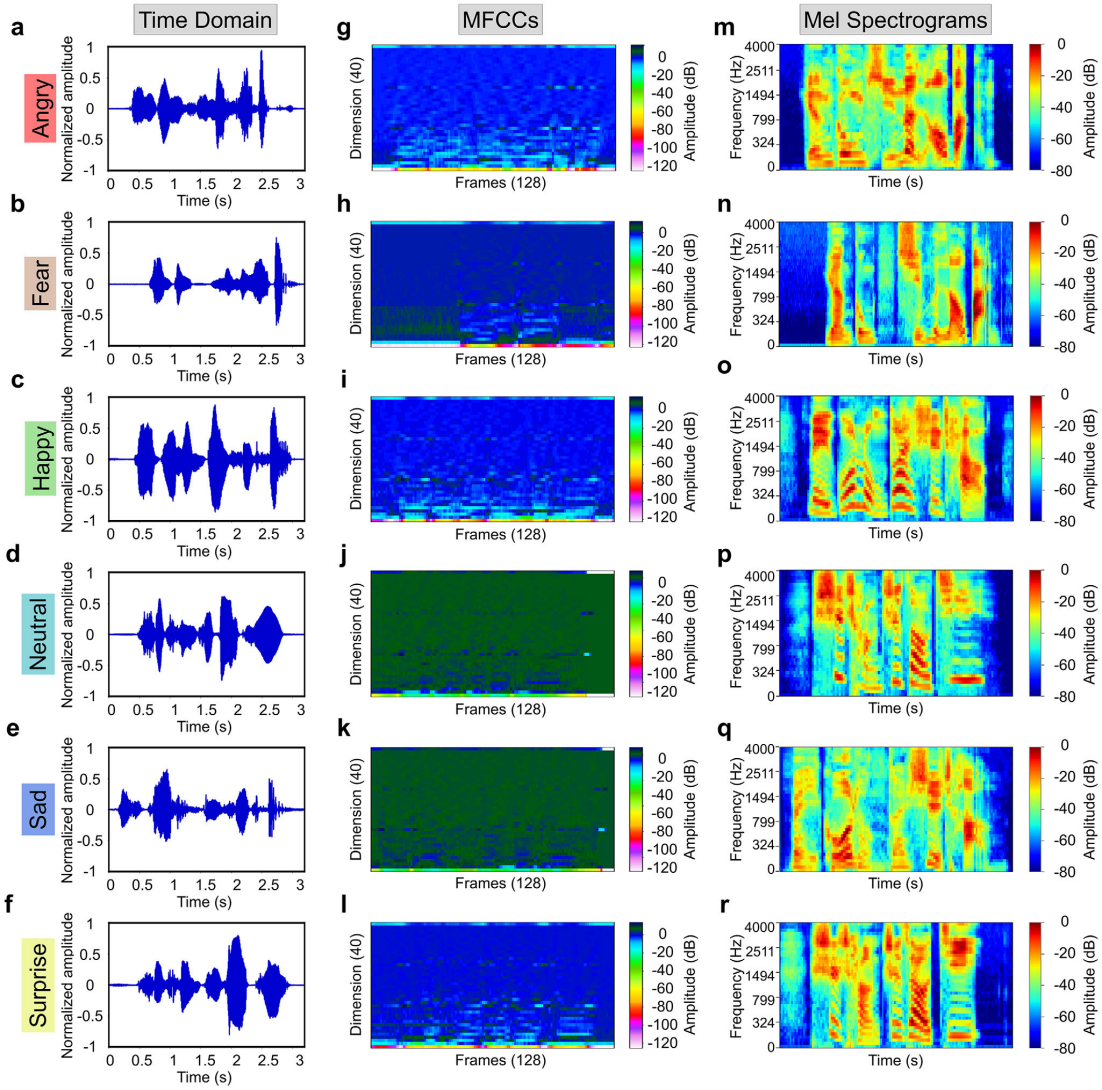
Signal processing. a–r) Time‐domain waveforms (a–f), Mel‐frequency cepstral coefficients (MFCCs) (g–l), and Mel spectrograms (m–r) of throat signals corresponding to six emotional states, including anger, fear, happy, neutral, sad, and surprise, while vocalizing the same sentence.

After validating the recording functionality of the ATPS, we then used our system to record 1200 speech signals from 4 subjects with six different emotional states. To enhance the discriminability of acoustic features, these signals were subjected to standardized preprocessing steps, including pre‐emphasis, framing with windowing, and endpoint detection (Note S2). In this study, multi‐dimensional features such as time‐domain waveforms, Mel Frequency Cepstral Coefficients (MFCC), and Mel spectrograms were extracted (Figure 5). The results demonstrated significant variations in energy profiles, frequency contents, and rhythmic patterns between emotions, with strong intra‐emotion consistency across all feature dimensions. For example, angry speech segments displayed significantly larger waveform amplitudes (Normalized Average Amplitude, ∼±0.25), greater time‐domain variability (Peak‐to‐Peak Normalized Amplitude, ∼1.55), increased speech rate (∼180 words/min), reduced pausing frequency (∼2.8 pauses/min), and a distinctly high‐energy acoustic signature (energy mean, 0.25; standard deviation, 0.12) compared to other emotions (Figure 5a). The representative MFCC features extracted from angry speech exhibited pronounced dynamic variations (ΔMFCC = 1.8; standard deviation = 1.4), spanning a broad frequency range (800–4000 Hz), while displaying distinct textural patterns and robust rhythmic signatures. And the Mel spectrogram indicated that energy speech primarily concentrated in the low‐frequency region (100–500 Hz) and mid‐to‐high frequency bands (1500–4000 Hz), reflecting the intensity and continuity of the Angry emotion. In contrast, sad speech exhibited significantly reduced time‐domain amplitude (Normalized Average Amplitude, ∼±0.08), slower speech rate (∼100 words/min), and more frequent pauses (∼6.5 pauses/min) compared to neutral speech, with overall energy level cut in half (Figure 5e). The representative MFCC features exhibited gradual changes with smooth voiceprint, presenting a low‐energy, low‐frequency, and relaxed rhythmic pattern. And the Mel spectrogram's energy was mainly concentrated in the low‐frequency region (100–500 Hz), with almost no high‐frequency components. Basically, while these features were multidimensional and quantitative, such preprocessing steps provided a vast of fingerprints for each emotional speech segment (Table S5).

**FIGURE 6:**
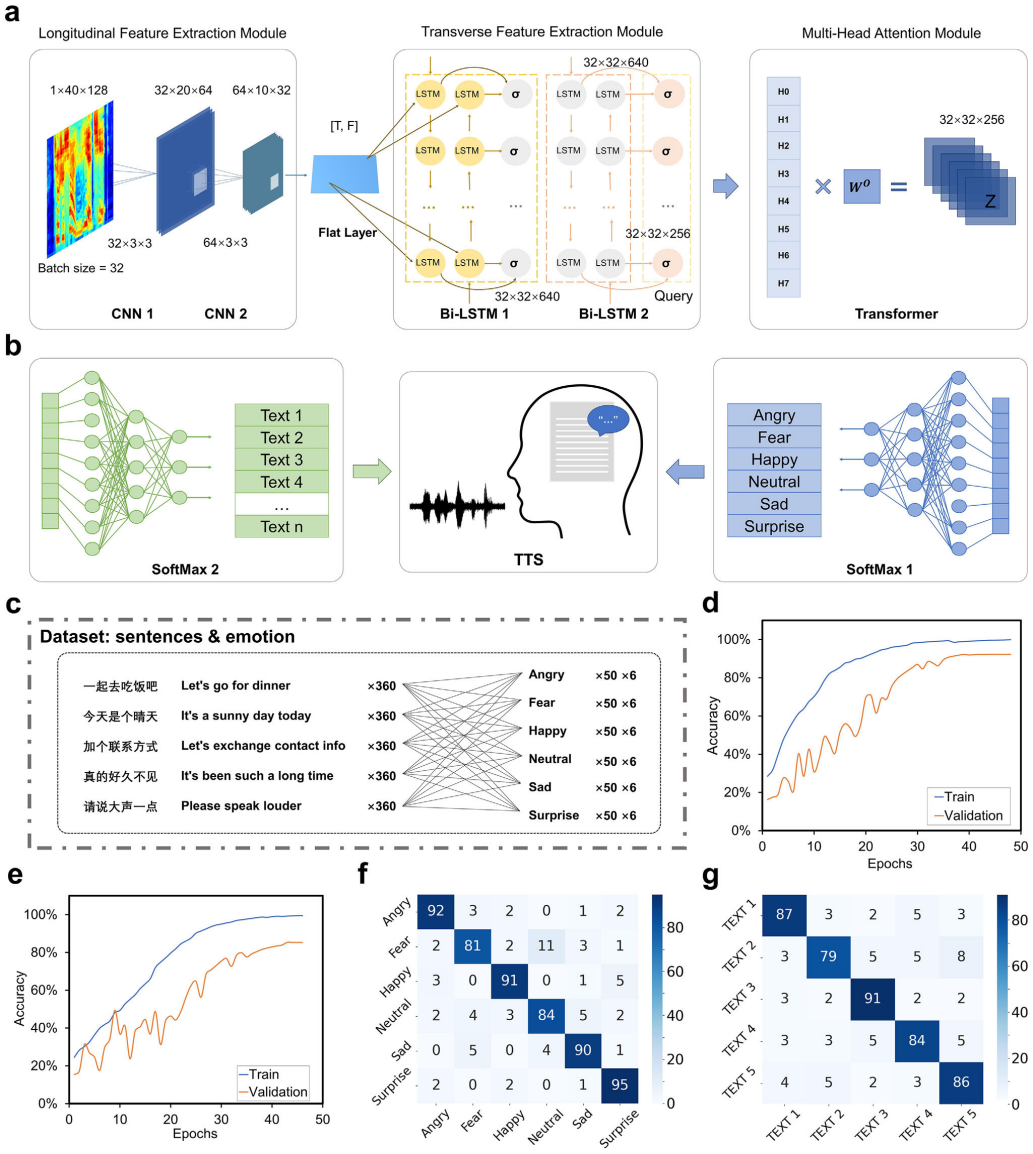
Emotive speech recognition through deep learning. a) Schematic of the integrated deep learning model (CNN‐LSTM‐Transformer), illustrating feature extraction and multi‐head attention mechanism design. b) Schematic diagram illustrates the process of classifying emotions and text through Softmax, and synthesizing emotive speech based on the TTS (Text‐to‐Speech). c) Distribution of the dataset used for model training, including 5 different sentences from various texts and 6 different emotional categories. d,e) Training and validation accuracy versus epochs for the (d) emotion recognition model (overall accuracy of 90.20%) and (e) text recognition model (overall accuracy of 84.71%). f,g) The confusion matrix for the (f) emotion recognition results and (g) text recognition results of the test‐set cover 6 different emotion categories and 5 different sentences from different texts.

To further distinguish the intrinsic differences between the emotional information‐rich fingerprints, a deep learning‐based recognition and classification method was employed to define functional clusters of the features (Figure 6). Figure 6a illustrates the architecture of our proposed deep learning framework, comprising three key components: (1) Initial CNN layers that extract local time‐frequency patterns from MFCC features through hierarchical convolution operations; (2) Bidirectional LSTM (Bi‐LSTM) layers for modeling both forward and backward temporal dependencies in the sequence; and (3) A Transformer layer employing multi‐head self‐attention mechanisms to capture long‐range contextual relationships across the entire signal. To recognize and classify the emotional speeches, two classifiers (FC Layer + SoftMax) were implemented to simultaneously identify the texts and emotions of the speeches in this study (Figure 6b). A TTS‐based method coupling with a user interface was also employed here to enable final synthesis of emotional speeches (Figure S17). 6 emotional categories (Angry, Fear, Happy, Neutral, Sad, Surprise) and 5 different text sentences (“Let's go eat”, “Today is a sunny day”, “Add me on contact”, “Long time no see”, “Please speak louder”) from 4 individuals, were recorded and tested using our platform for further model training (Figure 6c). To prevent the model from overfitting to individual subjects, we recruited participants of varying genders and age groups. During model training and evaluation, we strictly adhered to a “leave‐one‐subject‐out” cross‐validation scheme. For the four subjects included in this study, we conducted four‐fold cross‐validation: each time data from one subject were held out as the test set, while data from the remaining three subjects were used for training. Average emotion recognition accuracies were 87.83%, 89.50%, 91.00%, and 89.17%, respectively; and average text recognition accuracies were 80.4%, 81.6%, 79.4%, and 80.2%, respectively (Figure S18). As shown in Figure S19, the validation loss plateaued without a subsequent increase, and the validation accuracy stabilized closely with the training accuracy. Collectively, these results indicated no evident sign of overfitting during training and a highly stable generalization of our model across subjects. The finalized deep learning model achieved classification accuracies of 90.20% for emotion recognition (Figure 6d) and 84.71% for text recognition (Figure 6e) using MFCC‐based features. The confusion matrices quantitatively exhibited the model's classification performance across emotion and text categories of another two subjects (independent tests), revealing 88.83 ± 5.27% and 85.40 ± 4.39%, 89.00 ± 3.41% and 85.40 ± 3.36% in accurate classifications, respectively (Figure 6f,g; Figure S20). These results demonstrated the model's effectiveness in jointly modeling emotional and lexical content from the signals recorded via the ATPS. Importantly, due to the anti‐interference characteristics of the sensor and the decoupling ability of our AI model, typical motion artifacts or environmental noises are not likely to significantly interfere with ATPS (Figure S21). Taken together, these results demonstrated the potential utility of the ATPS in emotional speech recognition.

## 3 Discussion

In this study, we demonstrated emotional speech recognition by combining a wireless, battery‐free throat patch system and deep learning algorithms. Previous strategies for speech recognition have primarily focused on lexical content reconstruction (e.g., words or sentences), but exhibit fundamental limitations in capturing paralinguistic features such as speaker‐specific timbre or emotional prosody [16, 17, 18, 19, 20, 21]. Meanwhile, recent advances in artificial throat systems have demonstrated the capability to both detect and synthesize acoustic signals by decoding information‐rich throat movements, offering a promising alternative to conventional speech recognition paradigms [21]. However, current artificial throat systems suffered from several critical limitations, including tethered operation, mechanical rigidity, and restricted sensing bandwidth, which significantly hindered their practical deployment in wearable devices for mute individuals [18, 19, 20, 21]. To overcome these limitations, we proposed an ATPS, comprising a thin‐film strain sensor integrated with a miniaturized FPCB for sensing the throat signals with a relatively broad frequency spectrum. Energy harvesting and wireless data transmission were simultaneously accomplished through NFC coupling between a smartphone and the FPCB antenna. This battery‐free, light‐weighting design eliminated the need for external power sources and reduced system bulkiness, resulting in a more compact and user‐friendly wearable patch particularly suitable for disabled individuals. Furthermore, the system incorporated deep learning algorithms to enable emotion recognition from throat signals. Functional validation experiments also indicated that the platform achieved high classification accuracy for both emotional states (e.g., happiness, anger, sadness) and lexical contents. Collectively, compared to other laryngeal patches, the advantages of our device lied in the full integration and the recognition functionality. To our best knowledge, the ATPS is the first patch capable of simultaneously performing both speech and emotion recognition (Table S6).

As a proof‐of‐concept study, we have demonstrated the functionality of the ATPS for the emotional speech's recognition. It is noteworthy that this system holds potential for translation to various other bio‐applications, such as functional monitoring and intelligent analysis of heart, blood pressure, and sleep breathing [58, 59, 60]. Indeed, several areas for further improvement exist: 1) The thickness of the encapsulated device appears to be ∼2.5 mm, mainly resulting from the sensing layer with the waterproof polyurethane film (∼750 µm) and the encapsulated device (∼1.75 mm), which could be further miniaturization include optimizing the elastomeric materials, micro‐structures, and thin‐film encapsulation. 2) As shown in Table S7, the elapsed time for generating emotional speech output involves six key phases, with a total typical latency less than 0.5 s. While such sub‐second latency is acceptable for natural conversation, particularly for remote communications, there is still considerable space for improvement. 3) As shown in Figure 3f, our device exhibited a non‐monotonic response in sensitivity tests across different frequency bands in air environment, with a notable decline primarily observed in both low‐ and high‐frequency ranges. The main reason might be that the mechanical vibration coupling itself was frequency‐dependent: even with a constant driving voltage, the effective vibration amplitude actually reaching the device varies with frequency (e.g., due to the acoustic source, coupling medium, and internal structure of the device) [61]. In the low‐frequency range, the radiation efficiency from the acoustic source to air decreases sharply. Changing the coupling medium to water or tissue environments have improved radiation efficiency, but a slight decline was still observed (Figure 3f). Besides, the dynamic mechanical impedance of the device stack (PDMS/fiber/microstructure) also varies with frequency, with the highest contact modulation efficiency achieved in the mid‐frequency range. However, sensitivity attenuation was observed in the high‐frequency regime, attributable to damping losses and the structural limitation of incomplete contact recovery between cycles [62]. In addition, small fluctuations of the sensing signals have been observed during cyclic testing (Figure 3h,i). It might stem from the interplay between the intrinsic physical properties of the piezoresistive materials and the testing environment. Due to the thermal motion of charge carriers, random voltage fluctuations would be generated, resulting in a naturally present broadband white noise baseline in the piezoresistive material during testing [63, 64]. Furthermore, under cyclic stress, non‐uniform polymer substrates or composite materials would exhibit time‐dependent relaxation in the response of their molecular chains or internal interfaces, leading to a non‑instantaneous relationship between resistance change and applied stress, thereby introducing hysteresis and fluctuations [63, 64, 65]. Therefore, integrating more advanced microstructures or materials with enhanced uniformity, improving system isolation and packaging would significantly enhance the sensitivity and stability of throat signals detection in mute people. 4) Additionally, multiple further in‐depth studies, including inputs from more subjects with more speeches, real‐time recognition, privacy protection, and clinical tests, were also warranted. And the overall recognition accuracy can be refined by taking advantage of more advanced deep learning algorithms and appropriate sensitivity correction of the datasets covering wide range of frequencies. We believed that our ATPS platform holds significant potential as a foundational technology for emotional speech recognition, and establishes a novel paradigm for personalized assistive communication, particularly for patients with vocal cord disorders or laryngeal impairments.

## 4 Experimental Section

### 4.1 Fabrication of CNT/PDMS Layer

MWCNTs (C742308, Macklin) were dispersed in isopropanol (I811932, Macklin) at a mass ratio of 1:15. The mixture underwent ultrasonic dispersion for 2 h, followed by vacuum drying (60 °C, 4 h) and sieving (200‐mesh sieve) to obtain modified MWCNTs powder. The modified MWCNTs were blended with PDMS matrix loading. After degassing, the composites were spin‐coated onto photolithographic molds, followed by thermal curing at 80 °C for 2 h (Figure S22).

### 4.2 Fabrication of MWCNTs/EC Layer

A 10 wt.% EC (270–330 mPa·s, Aladdin) solution was prepared by dissolving EC in a binary solvent mixture of Tetrahydrofuran (THF) and N,N‐dimethylacetamide (DMAc) (1:1 v/v), and electrospun (YLKY‐2020A). After drying, the modified MWCNTs were uniformly spray‐coated onto the surface of the EC membrane. The composite membrane was then thermally pressed at 80 °C. Finally, plasma treatment (50 W, 120 s) was applied to further improve interfacial bonding (Figures S23 and S24).

### 4.3 Assembly of the Sensor

The MWCNTs/EC membrane was vacuum‐compressed and bonded between two CNT/PDMS films, then encapsulated with polyurethane protective films to obtain the artificial throat composite membrane. Electrical connections were established by curing Ag/AgCl conductive paste onto the copper wires to form the final sensor that used in the ATPS (Figures S25 and S26).

### 4.4 Simulation of the CNT‐PDMS Structured Films

Finite Element Analysis (FEA) of the CNT‐PDMS structured films was performed using COMSOL Multiphysics 6.0. The simulation incorporated experimentally determined material parameters (Young's modulus: 1.87 MPa, Poisson's ratio: 0.49), with fixed constraints applied at both ends. A static preload (0–60 mN) corresponded to the equivalent surface force derived from subglottal pressure and a dynamic load of 10×sin(400πt) mN superimposed on a 10 mN static pressure corresponded to the force transmitted through tissue resulting from vocal‐fold oscillation were applied to simulate laryngeal vibrations, enabling comparative analysis of the mechanical responses across different microstructure films. For fatigue‐life prediction, the static load was set at 50 mN, and a sinusoidal dynamic load with an amplitude of 10 mN and a frequency of 200 Hz was superimposed. The S‐N curve obtained from simulation was input as the boundary condition of the fatigue analysis (the S‐N curve was set as: from the 0.085 MPa load amplitude corresponding to 1.0e8 cycles to the 0.8 MPa load amplitude corresponding to 1.0e6 cycles curve) which defined the boundary conditions for the fatigue characteristics of the material. (Tables S1–S3 and Figure S5).

### 4.5 Characterization of the Sensor

The surface morphology was examined using SEM (MAGNA, Tescan). Mechanical properties were characterized via a computer‐controlled universal testing machine (0FBUTM6104, SANSIZONGHENG). Electrical resistance measurements were performed with a digital multimeter (DM3068, RIGOL). Sensor excitation was conducted on an acoustic platform with controlled sound pressure levels and frequency inputs. Properties of acoustic sensing were measured and analyzed by using an audio signal analyzer (AD2122, AOPUXIN) with a standard microphone. The microphone positioned 0.5 cm from the device functioned as a real‐time feedback sensor for sound pressure. This setup was implemented to ensure that the actual acoustic pressure applied to the device remained consistently close to the predetermined target value. To assess the sensor's performance consistency, we considered sample‐to‐sample and batch‐to‐batch variations. Sensors from three different batches, over a span of two months, were included in all tests to evaluate the reproducibility and stability of the sensor.

### 4.6 Fabrication of the FPCB

The PFCB was fabricated using a PI substrate with 1 Oz patterned copper traces, followed by surface deposition of a gold layer, achieving a final thickness of 200 µm. Lead‐free low‐temperature solder paste (LF 999, KELLY SHUN) was employed to assemble surface‐mounted components, including: 1) Microcontroller (STM32L051C8T6, STMicroelectronics); 2) Operational amplifier (OPA333, Texas Instruments); 3) NFC controller (ST25DV64K, STMicroelectronics);4) Power management IC (ME6211, MICRONE) 4) Various chip resistors and capacitors (Figure S10). The soldering process was conducted at 230°C. By utilizing the vector network analyzer (SNA5052X, SIGLENT) to optimize the coil tuning, its resonant frequency reached 13.56 MHz for stable NFC interaction (Figure S11). The peak data transmission rate based on the ISO 15693 communication protocol is 53 kbit/s.

### 4.7 Data Acquisition and Feature Extraction

The analog‐to‐digital conversion of the sensor signals was enabled by the FPCB at a sampling rate of 4 ksps with 12‐bit resolution, and the signal is compressed using the ADPCM (Adaptive Differential Pulse‐Code Modulation) algorithm. The acquired signals were processed through pre‐emphasis (coefficient: 0.98) (Note S2), followed by framing into 128 overlapping segments with Hamming windowing and endpoint detection. Feature extraction was carried out using Mel‐frequency cepstral analysis with a 40‐filter bank. The first 13 coefficients were selected as the primary feature parameters. These parameters were then augmented with the first‐order and second‐order differential parameters along with log‐frame energy values, resulting in a comprehensive 40 × 128‐D feature matrix for subsequent analysis (Note S3). The written consent of all the test contents from all participants was obtained prior to this study.

### 4.8 Data Set Acquisition

Before each recording, we guided subjects into a standardized emotional context. For example, to elicit anger, we asked the subjects to recall or imagine a real‐life event that had provoked intense anger and to describe its details and associated feelings as vividly as possible. For each target emotion, multiple standardized scenario scripts were provided to ensure consistency in the elicitation process. Subjects were then asked to read a standardized neutral text paragraph under each induced emotional state. This ensured that the linguistic content remained identical across different emotions, allowing acoustic differences to be primarily attributed to emotional variation. Besides, immediately after each recording, subjects completed a brief self‐assessment questionnaire, rating their emotional state along dimensions such as valence, arousal and dominance. Additionally, we randomly selected a subset of recordings for blind evaluation by independent reviewers who were not involved in the experiment. These reviewers categorized the perceived emotion. Only recordings for which both self‐reported ratings and third‐party evaluations showed significant agreement with the target emotion were retained for subsequent analysis.

The selected texts/words covered a balanced set of phonemes and syllable structures in Chinese and also included a variety of semantics used in daily communication (such as invitations, requests, commands, descriptions, and direct expressions of emotion). Though the texts/words used in the recognition might be a bit limited, it was crucial and sufficient for the preliminary testing of our method's speech recognition capabilities. All human trials involved in this work have been approved by the Seventh Affiliated Hospital of Sun Yat‐sen University (Shenzhen) Institutional Review Board (Approved No. Medical Ethics of KY‐2024‐037‐02).

### 4.9 Design and Experiments of Ensemble Algorithm

The proposed algorithm architecture integrated four key components (Note S4): a feature extractor (CNN), a temporal builder (LSTM), an encoder (Transformer), and a classifier (FC Layer + SoftMax). The model initially processed MFCC features through two CNN layers (3 × 3 kernels with 32 and 64 channels respectively, ReLU activation) to capture time‐frequency characteristics. Subsequent temporal dependencies were modeled using a bidirectional LSTM layer (2 layers, 128 units, dropout = 0.3), followed by a Transformer encoder (8 attention heads, 128‐D) for global context representation. The classification module employed global average pooling and a fully‐connected layer (128 units, dropout = 0.5) to simultaneously perform 6‐class emotion recognition and 5‐class text classification. The model training was conducted on a proprietary dataset (900 data points) sourced from 3 subjects randomly split into training (80%) and validation (20%) sets. The test sets were collected from another subject, which contained 300 data points that were evenly distributed across both the emotion and text categories. Model performance was primarily evaluated using confusion matrices, supplemented with traditional metrics including accuracy and precision. The algorithm was trained using the Adam optimizer with a learning rate of 0.001. Batch normalization was applied during training (based on mini‐batches of 64 samples), and early stopping was implemented with a patience of 10 epochs (Note S5).

### 4.10 Statistical Analysis

The bar and curve graph data were presented as the mean ± standard deviation (SD). A two‐tailed paired student *t*‐test or an independent samples *t*‐test was used to calculate differences between the two groups. Differences were considered statistically significant at ^*^ *p *< 0.05, ^**^ *p *< 0.01, and ^***^ *p *< 0.001. Unless otherwise specified, the data shown are means ± SD. No blinding was used.

## Author Contributions

X.L. conceived and coordinated the project. B.X., G.L., and X.H., designed and fabricated the ATPS, performed all the validation tests. K.J., J.Y., L.Z., X.H., and X.Q. assisted the data analysis. B.X., G.L., C.L., Y.Q., J.Y., and X.L. wrote the manuscript.

## Conflicts of Interest

The authors declare no conflicts of interest.

## Supporting information




**Supporting File**: advs74108‐sup‐0001‐SuppMat.docx.

## Data Availability

The data that support the findings of this study are available from the corresponding author upon reasonable request.
