# Supplementary material for: A Wireless, Battery‐Free Artificial Throat Patch with Deep Learning for Emotional Speech Recognition
Source: Adv Sci (Weinh). 2026 Jan 28;13(19):e16617. doi: 10.1002/advs.202516617 (PMC13045234; doi:10.1002/advs.202516617)
Supplement: Supplementary file 1 — Supporting File: advs74108‐sup‐0001‐SuppMat.docx. [file ADVS-13-e16617-s001.docx]

**Supplementary Information**

**A Wireless, Battery-Free Artificial Throat Patch with Deep Learning for Emotional Speech Recognition**

Bingxin Xu^1,3^, Guanming Lin^1,3^, Xiaohui Hong^1,3^, Chuting Liu^1^, Xinyi Qu^1^, Kangjian Jiao^1^, Lei Zhang^1^, Bingzhe Xu^1*^, Jun Yan^2*^, Yancong Qiao^1*^, Xudong Lin^1*^

^1^Guangdong Provincial Key Laboratory of Sensor Technology and Biomedical Instrument

School of Biomedical Engineering

Shenzhen Campus of Sun Yat-Sen University

Shenzhen, 518000, China

^2^The Second Clinical Medical College,

Guangzhou University of Chinese Medicine,

Guangzhou, 510720, China

^3^These authors contributed equally to this work

*Correspondence should be addressed to

Dr. Jun Yan, [yanjun1989_happy@126.com](mailto:yanjun1989_happy@126.com) ;

Dr. Bingzhe Xu, [xubzh5@mail.sysu.edu.cn](mailto:xubzh5@mail.sysu.edu.cn) ;

Dr. Yancong Qiao, [qiaoyc3@mail.sysu.edu.cn](mailto:qiaoyc3@mail.sysu.edu.cn) ;

Prof. Xudong Lin, [linxd37@mail.sysu.edu.cn](mailto:linxd37@mail.sysu.edu.cn) .

**Supplementary Tables**

**Supplementary Table S1. Detailed geometrical parameters of specific microstructures used in the finite element analysis.**

**
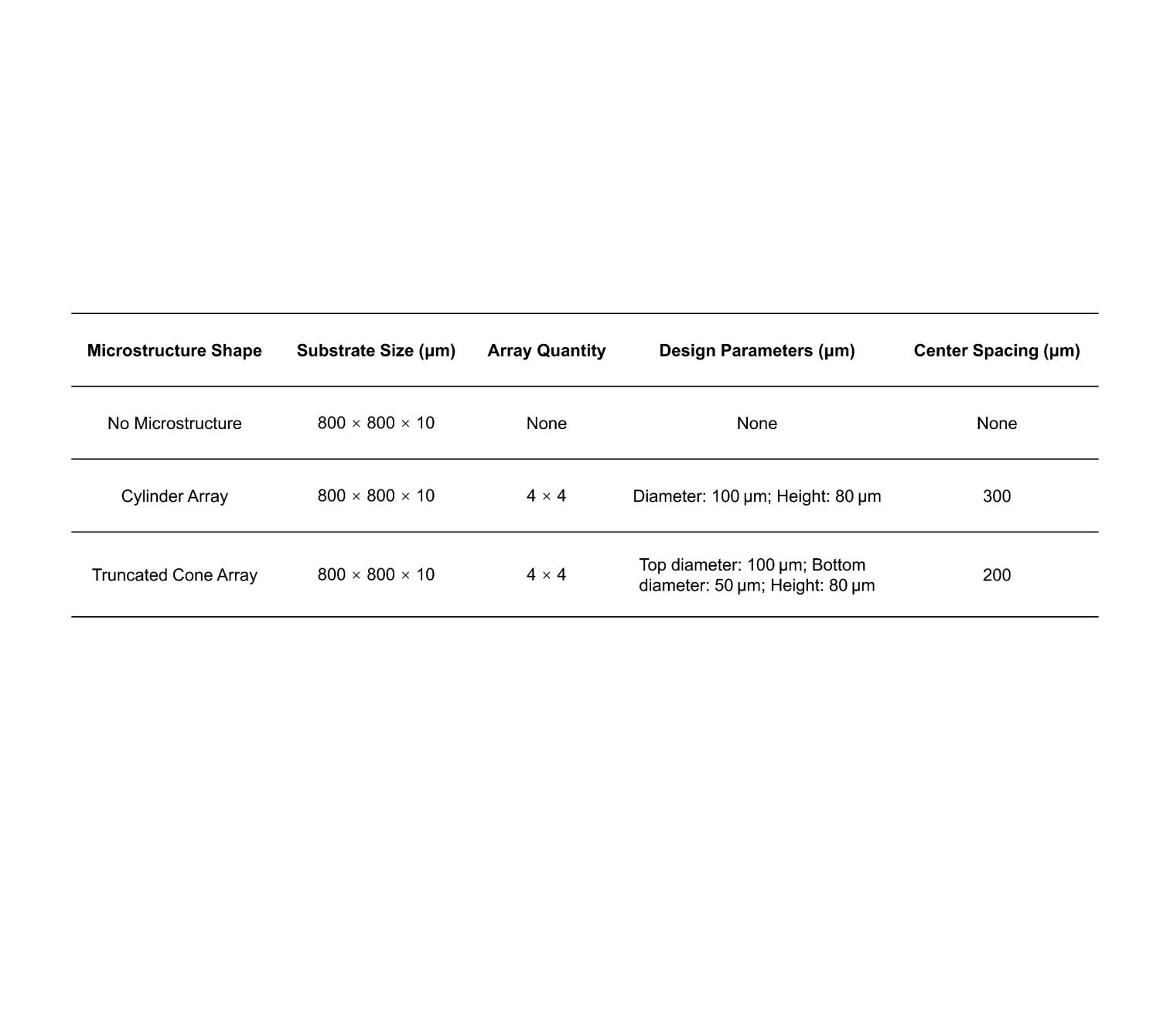
**

**Supplementary Table S2. Detailed geometrical parameters of three inverted truncated cone microarrays designed for finite element analysis.**

**
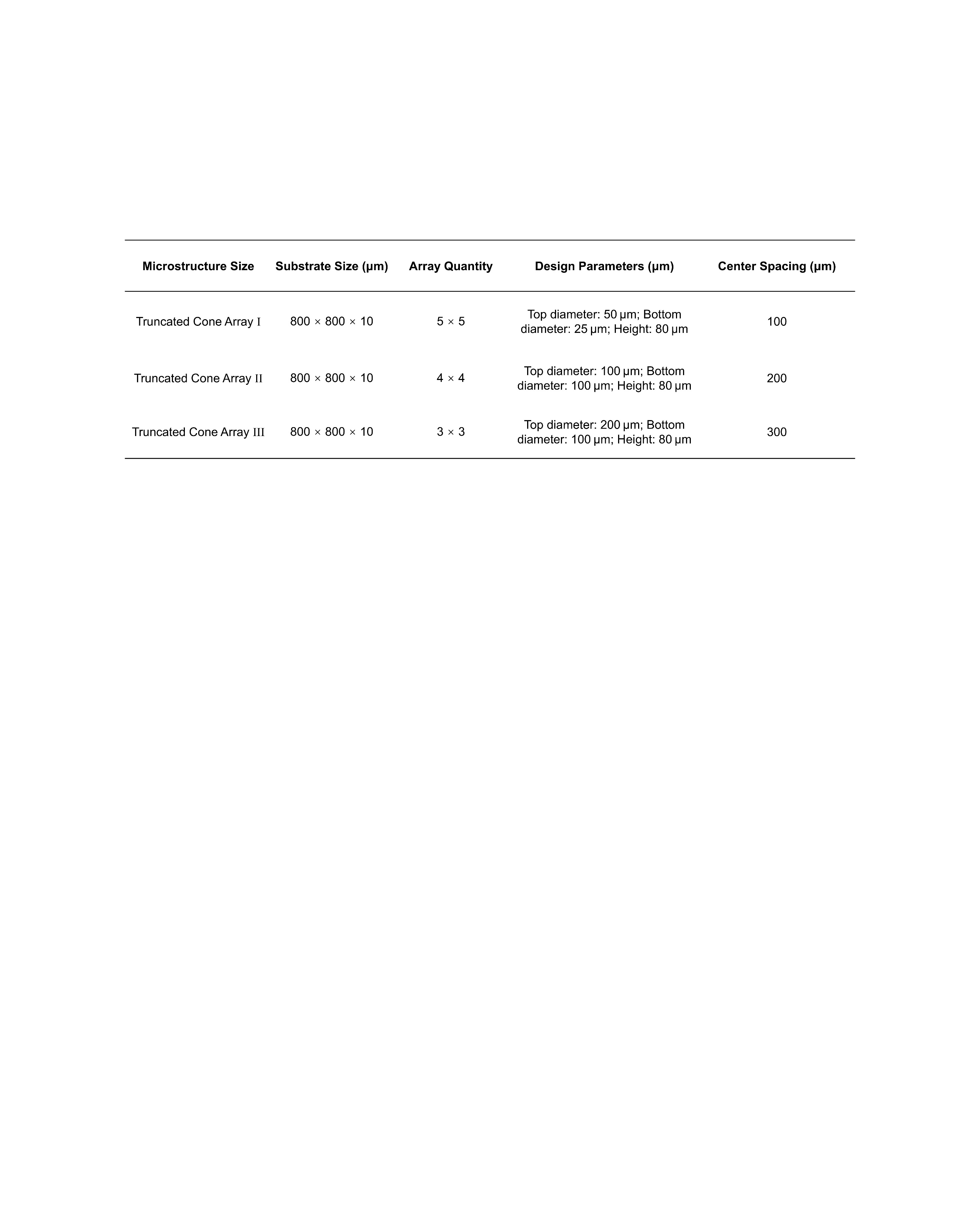
**

**Supplementary Table S3.** **Mechanical properties of CNT-PDMS films with different mass fractions of modified MWCNTs.**

**
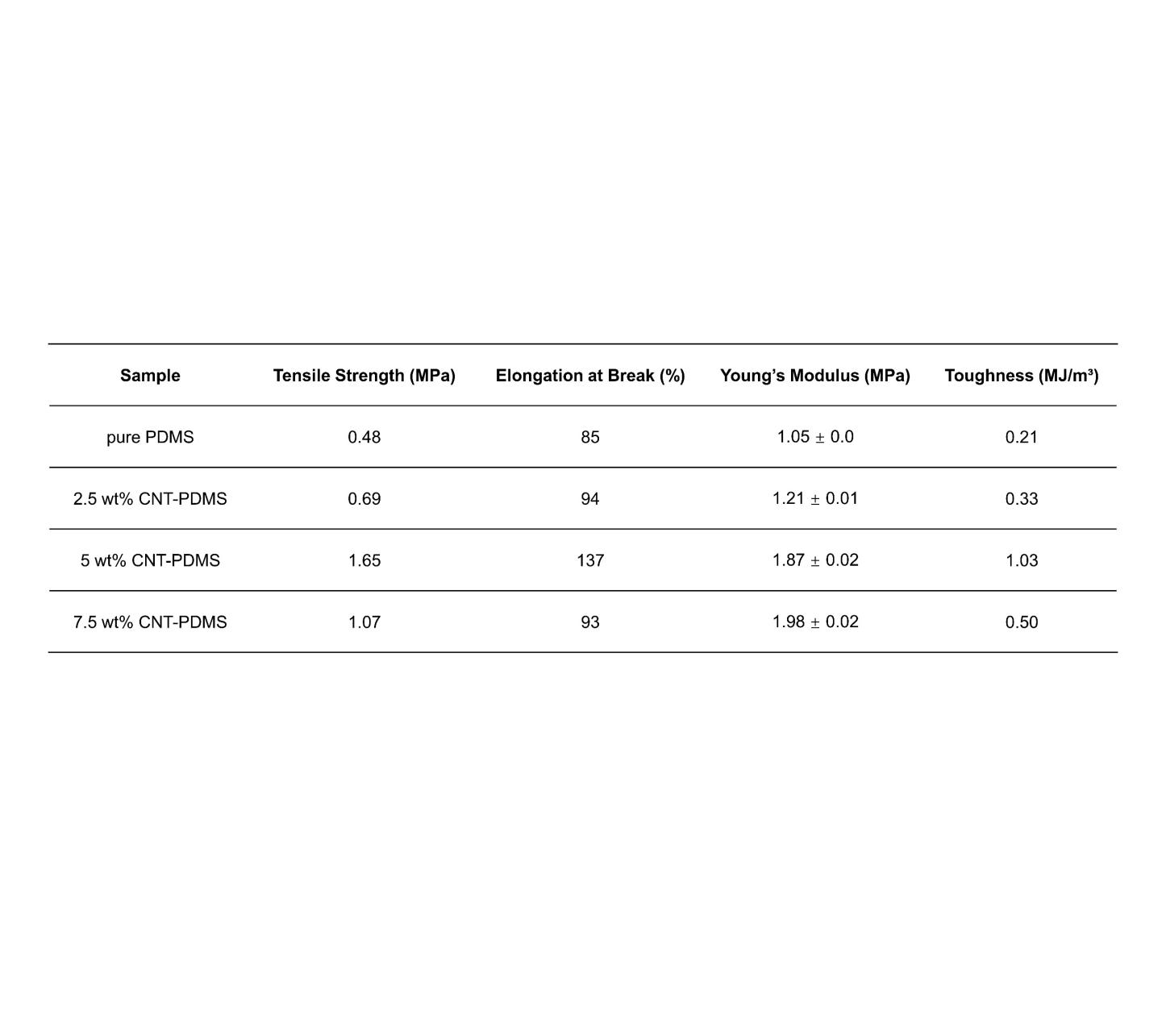
**

**Supplementary Table S4. Comparison of the reported strain sensors.**


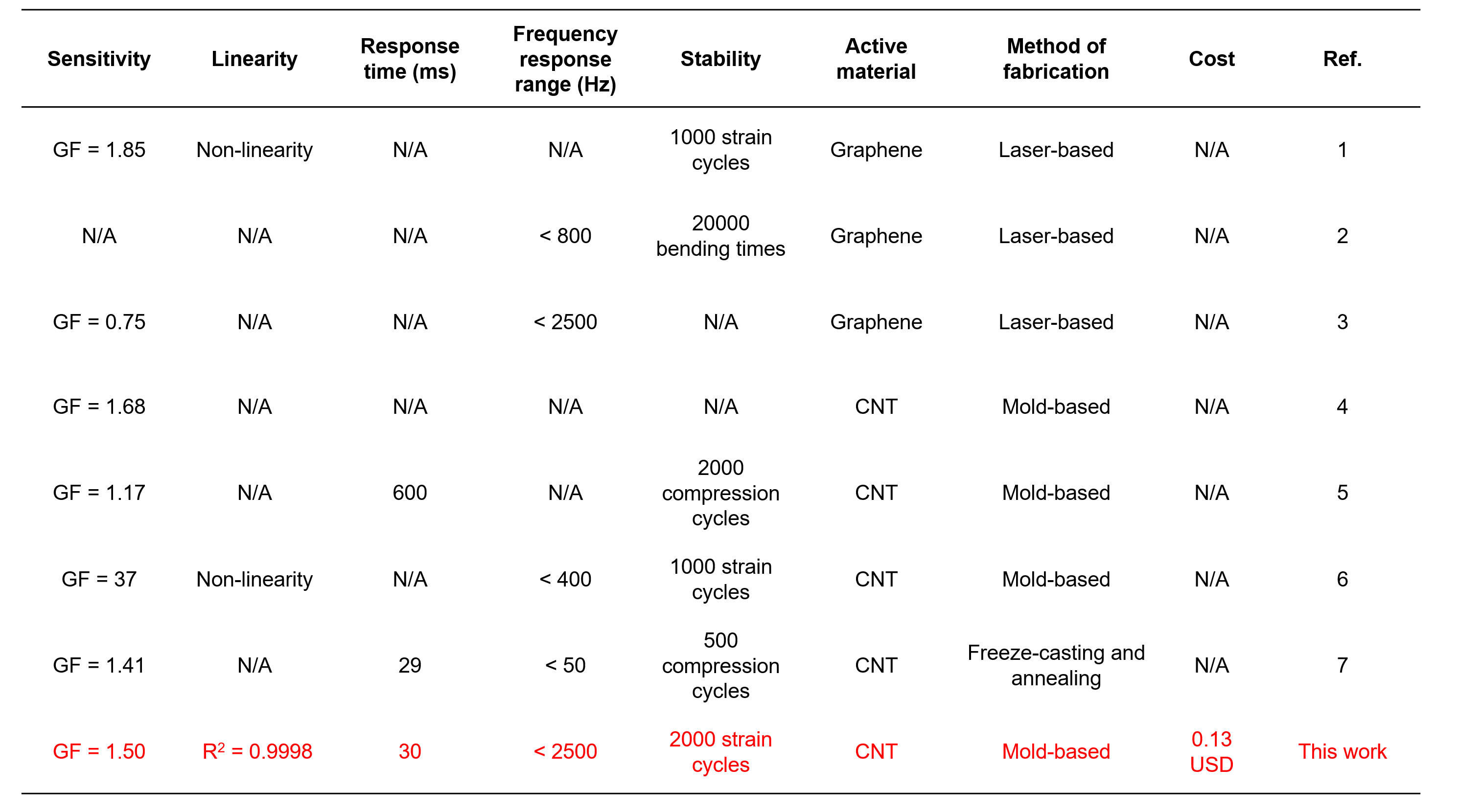


**Supplementary Table S5. The typical characteristics of different emotions in the Mel spectrograms.**

**
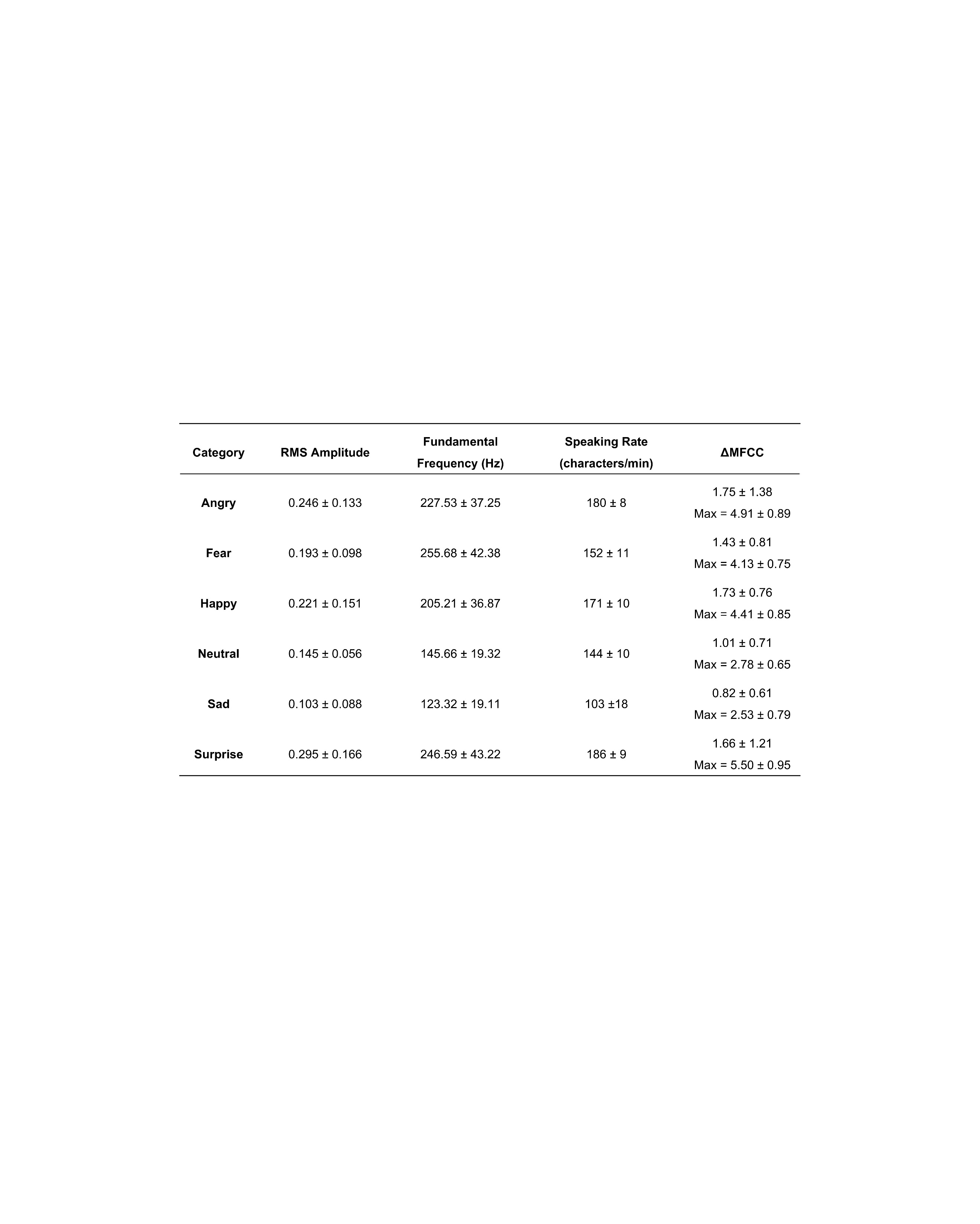
**

**Supplementary Table S6. Comparison of the reported wearable sensors for speech/emotion recognition.**

**
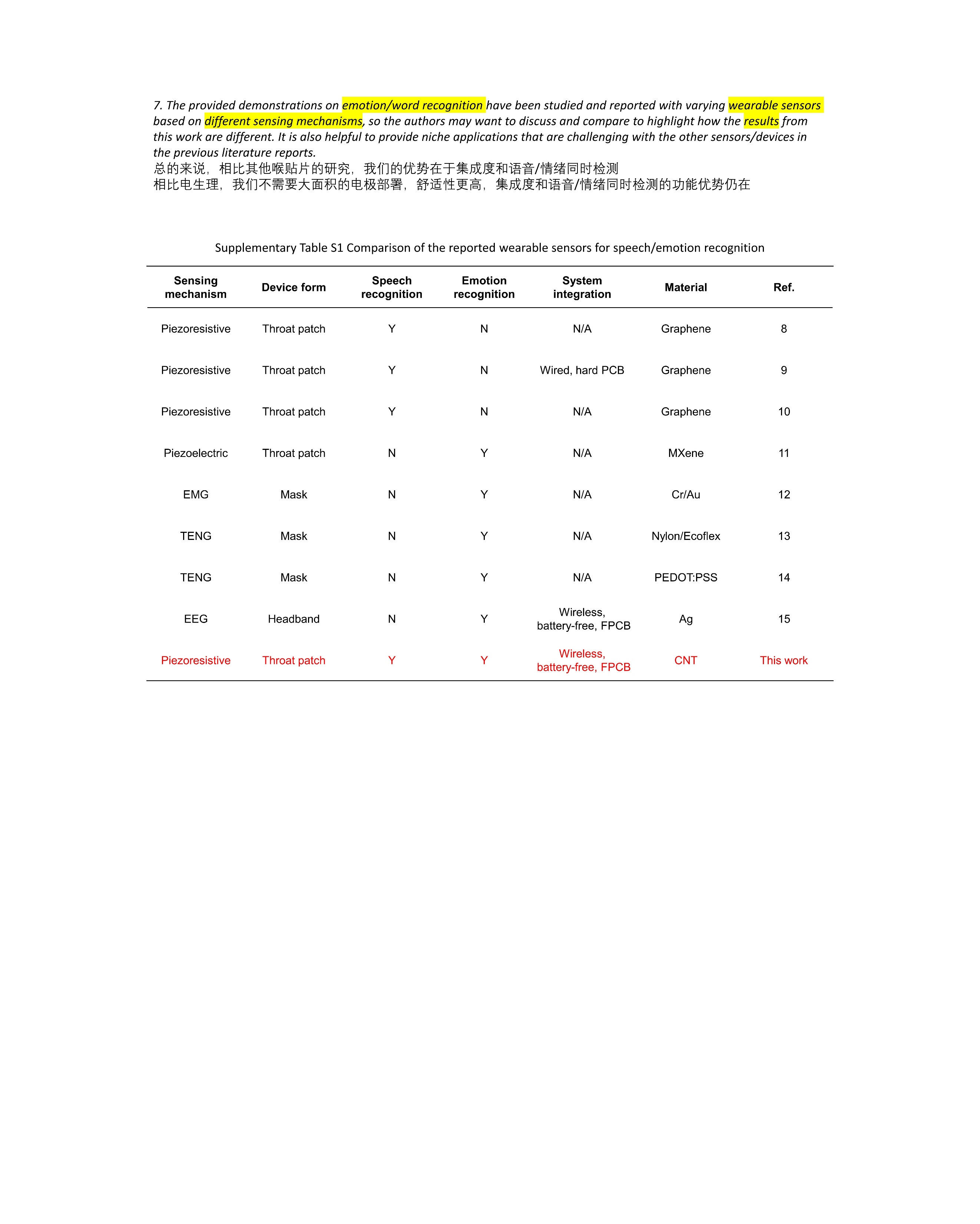
**

**Supplementary Table S7. The elapsed time of the complete signal processing pipeline in the ATPS.**

**
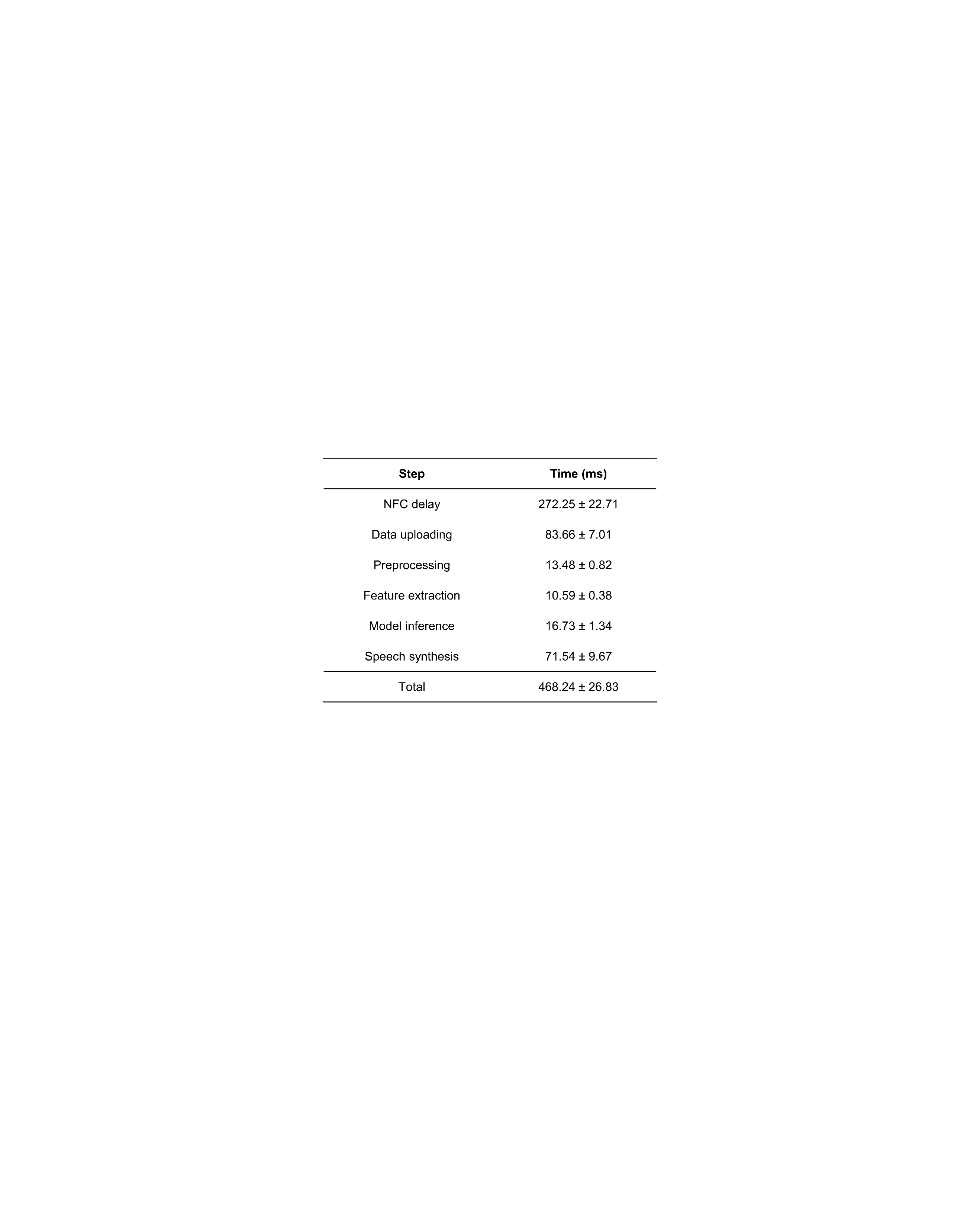
**

**Supplementary Figures**

**
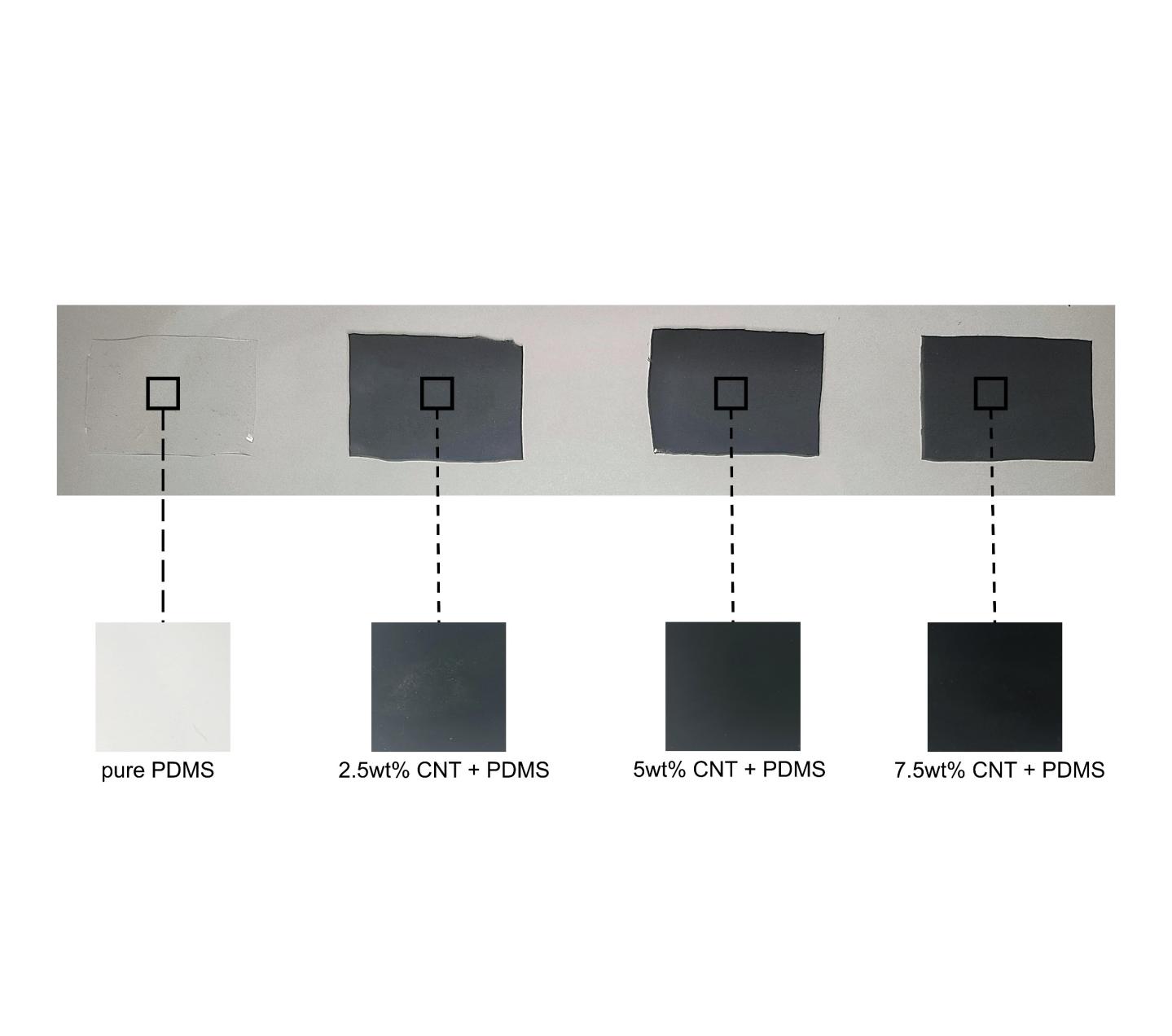
**

**Supplementary Figure S1.** **Photographs of CNT-PDMS film samples with varying mass fractions of modified MWCNTs.** With increasing MWCNTs loading, the color of the films gradually changes from transparent (0 wt%) to dark gray (2.5 wt%), blackish gray (5 wt%), and eventually deep black (7.5 wt%).

**
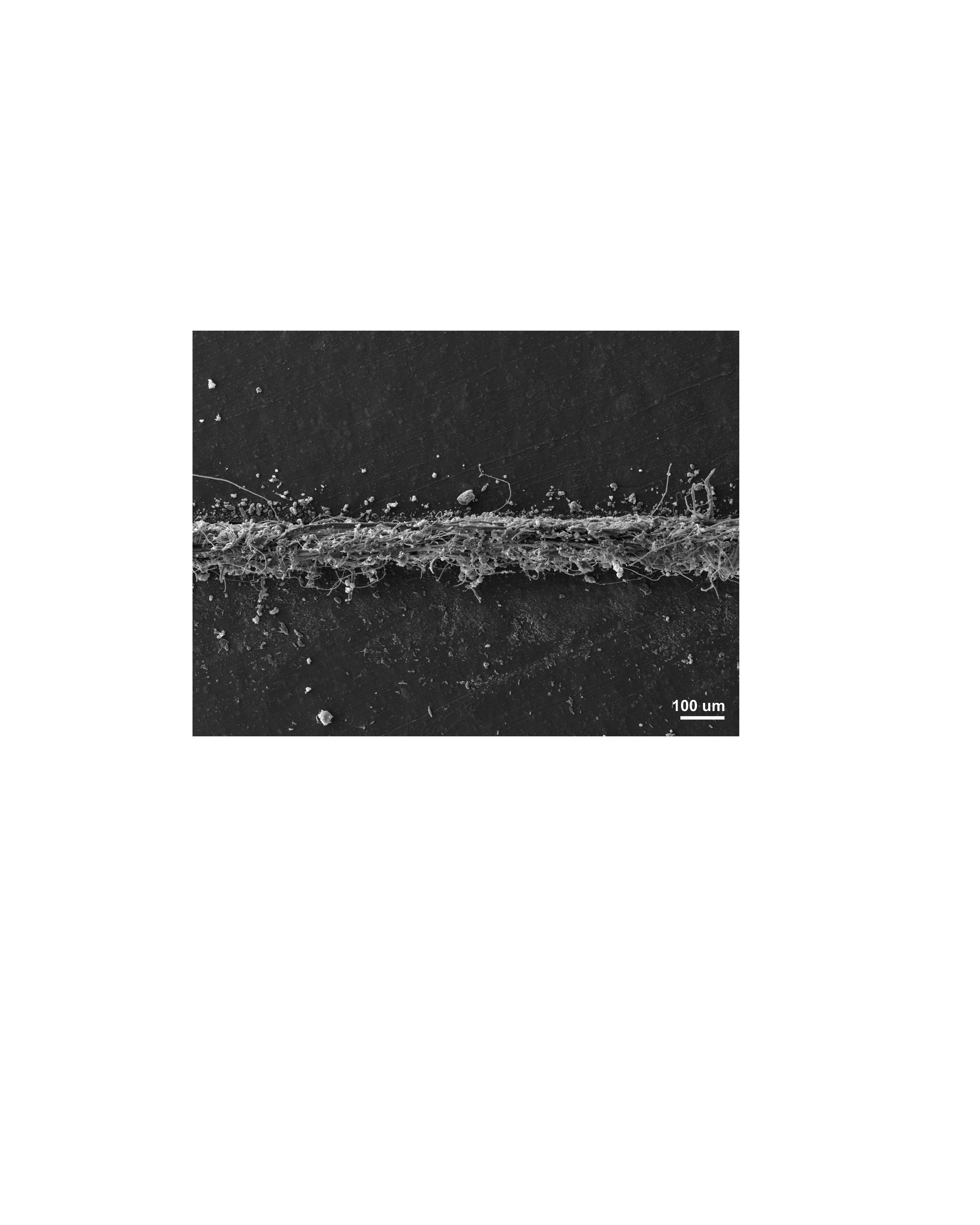
**

**Supplementary Figure S2. SEM images showing the cross-sectional view of the sensing film.**

**
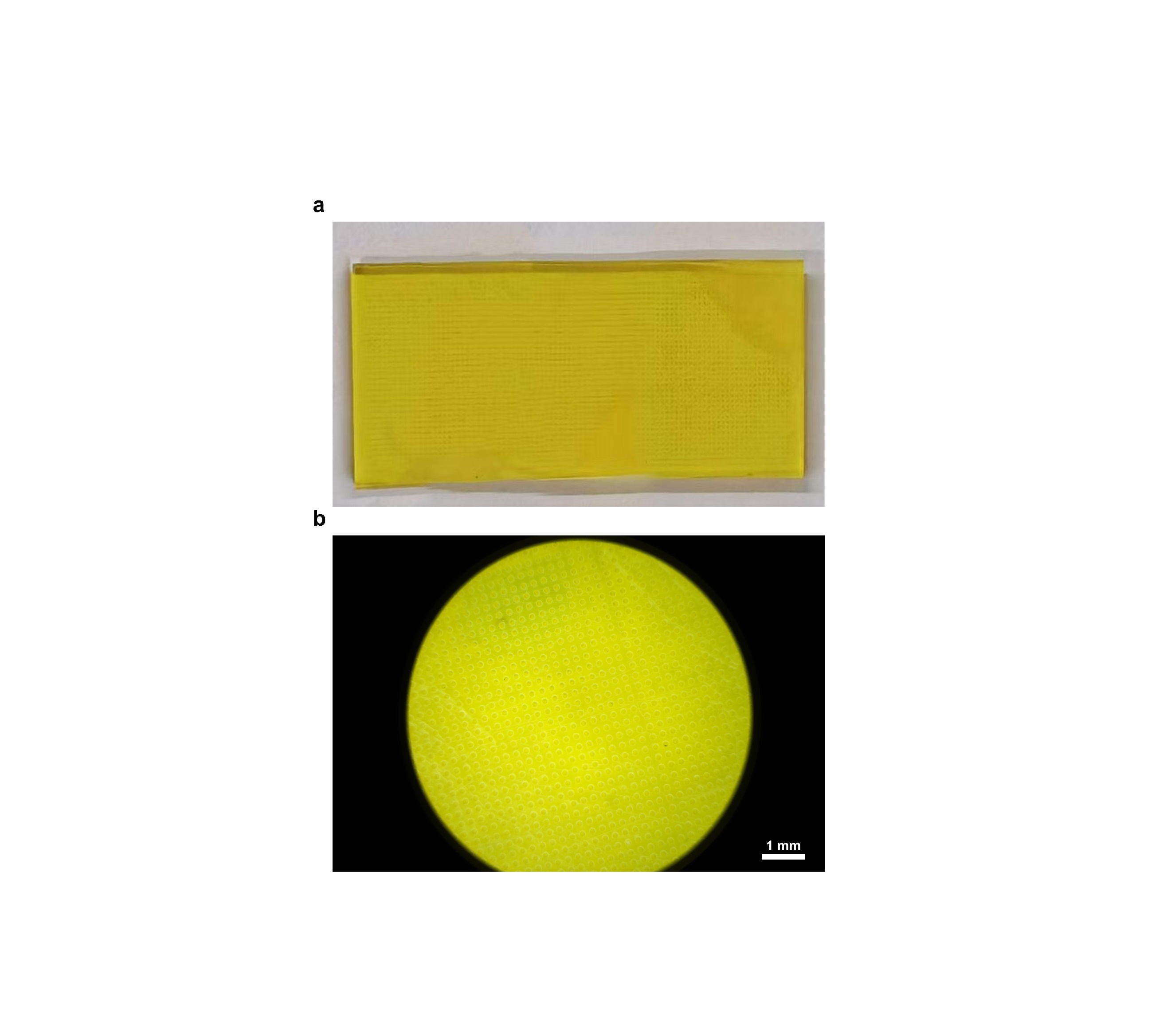
**

**Supplementary Figure S3. Fabrication of negative molds with inverted truncated cone microarrays.** a) Photograph of the printed mold. b) Inverted optical microscopy image of the mold. Scale bar, 1 mm. Microstructure array molds were fabricated using the nanoArch S140 micro-nano 3D printing system (Mofang Materials, Shenzhen; printing accuracy: ±10 μm).


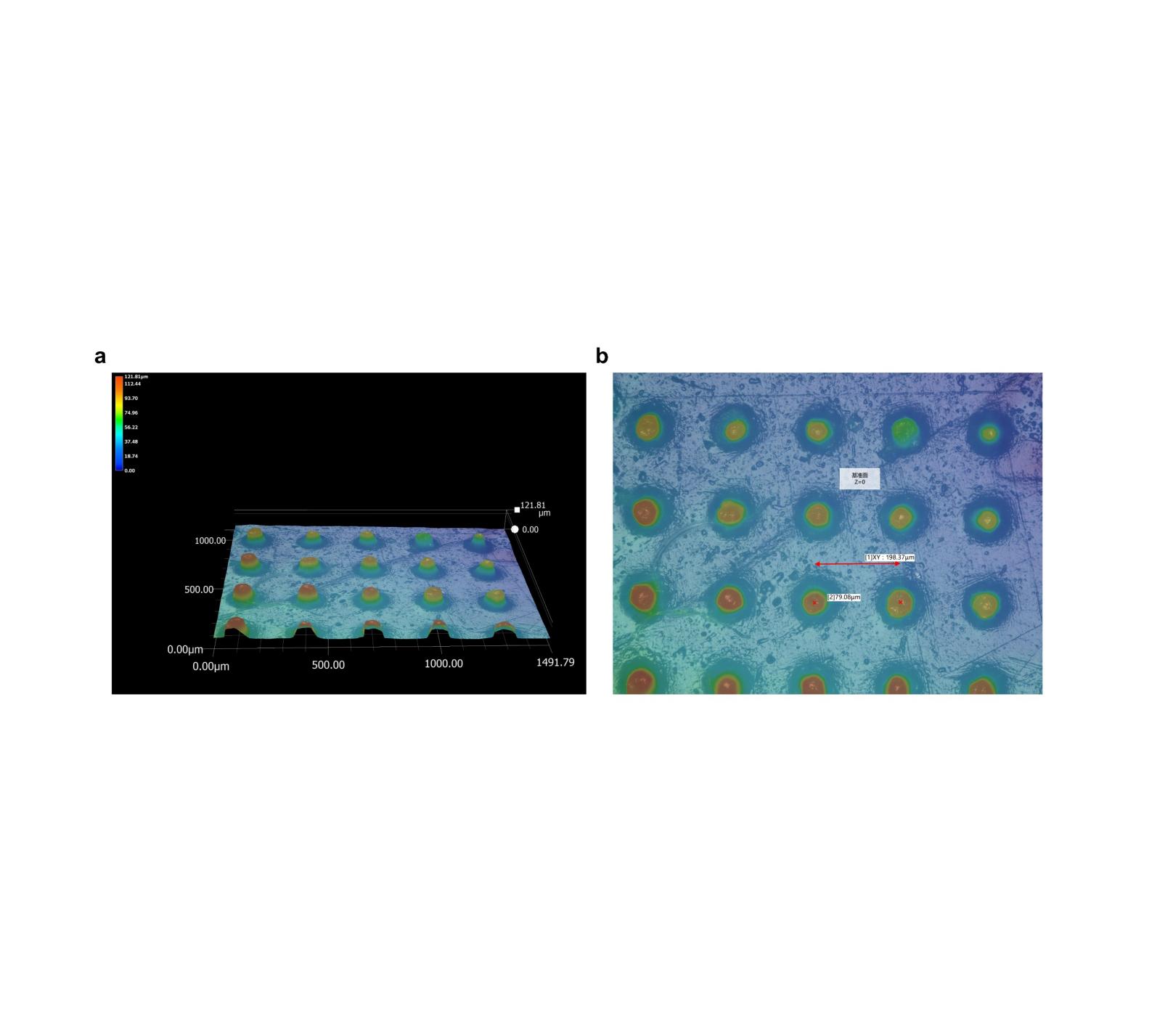


**Supplementary Figure S4.** **3D profilometry of CNT-PDMS II with micro-structured surface.** a) 3D reconstructed image of the CNT-PDMS film with microstructure arrays. b) Dimensional calibration of a representative inverted truncated cone microarray structure, showing a measured height of approximately 79.0 μm and a center-to-center spacing of about 198.37 μm. The overall fabrication error of the microstructured film is within 1.2%.

**
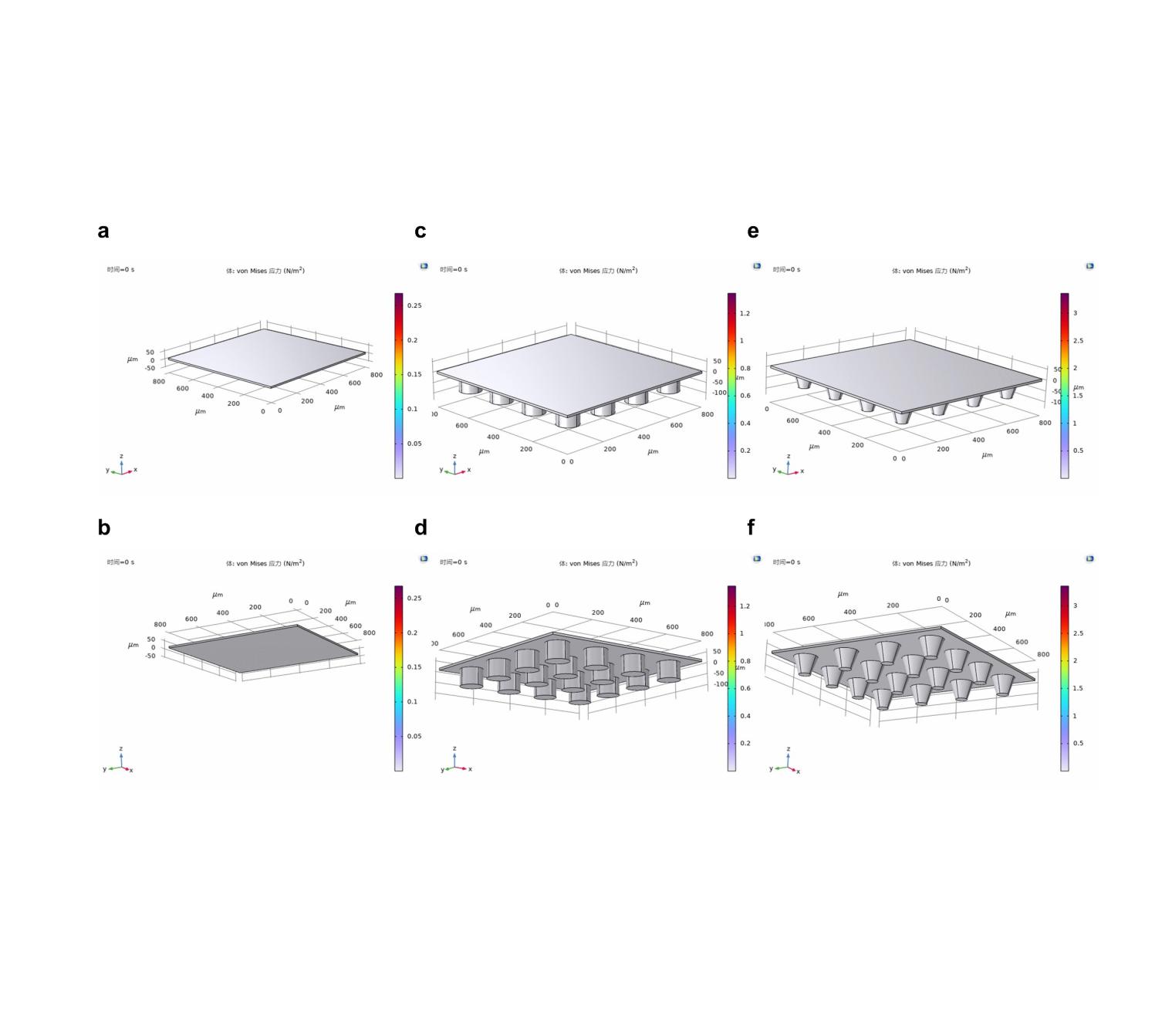
**

**Supplementary Figure S5.** **Stress-strain distribution in CNT-PDMS films with different microstructures.** a, b) Oblique view of CNT-PDMS film without microstructures. c, d) Oblique view of CNT-PDMS film with cylindrical microarrays. e, f) Oblique view of CNT-PDMS film with inverted truncated cone microarrays.

**
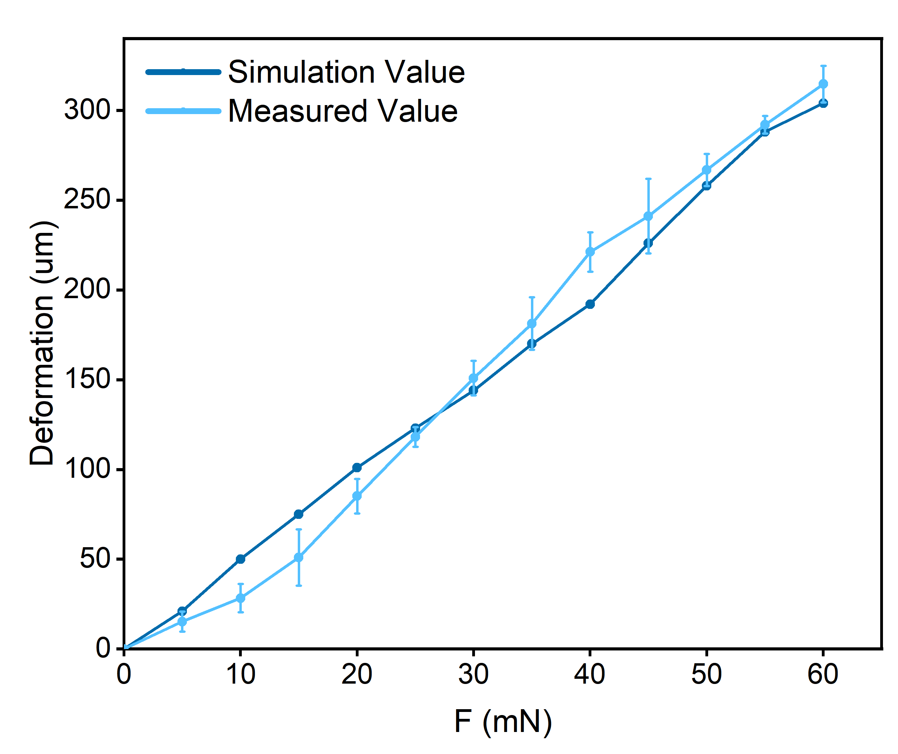
**

**Supplementary Figure S6. Comparison of simulated and measured load-dependent deformation of CNT-PDMS films.**


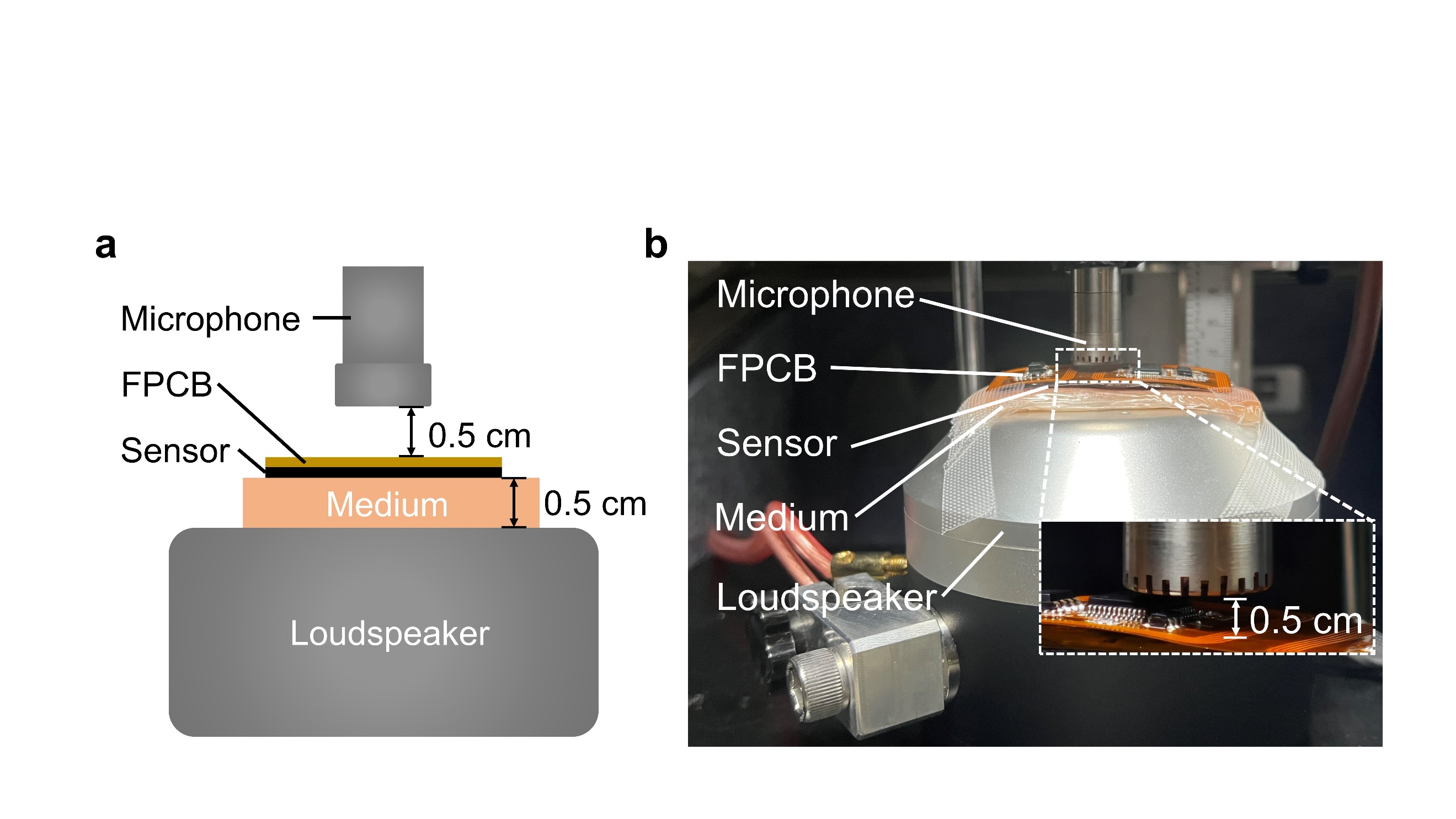


**Supplementary Figure S7. Acoustic testing platform.** Schematics (a) and photograph (b) of the acoustic testing platform for characterization of sensor.

**
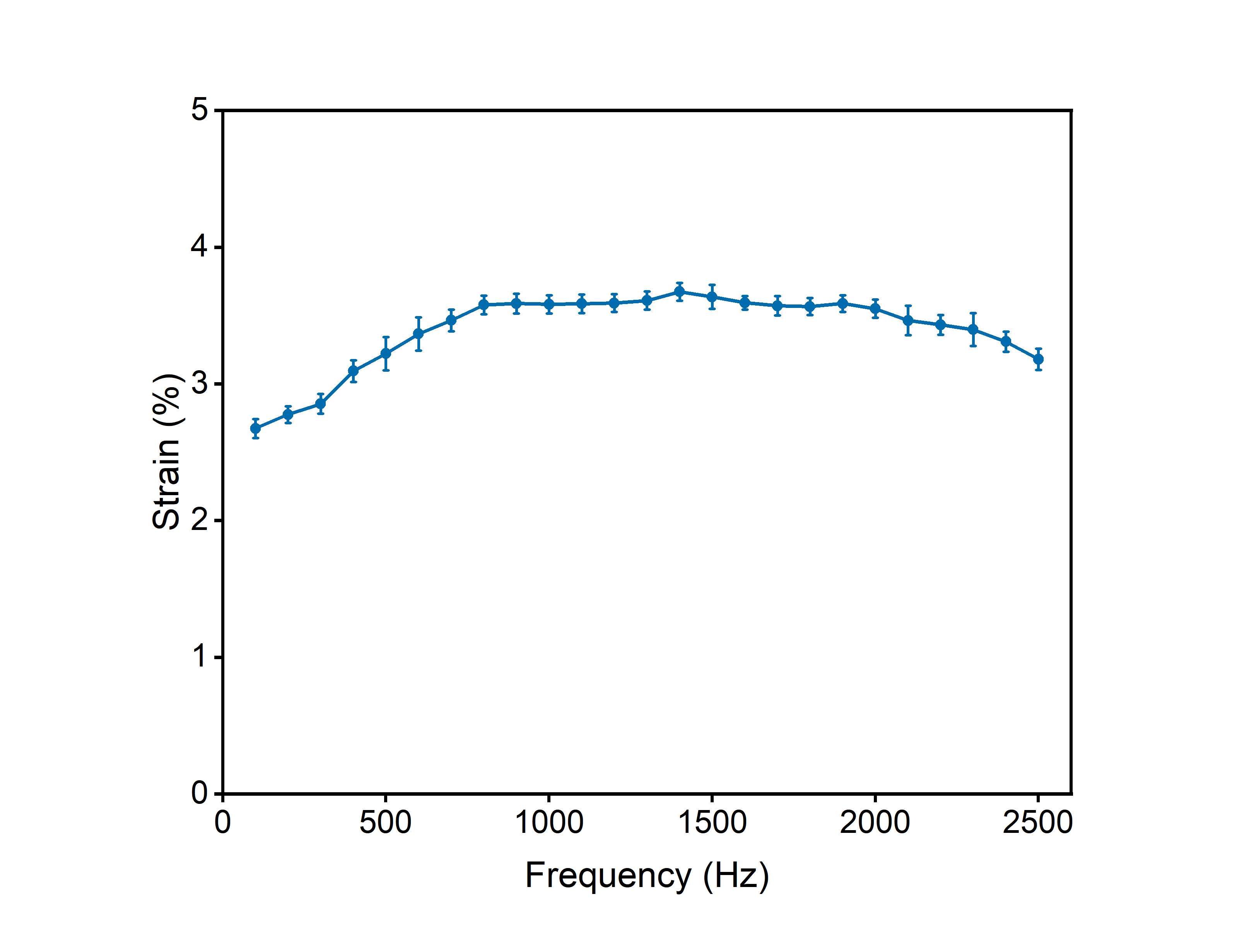
**

**Supplementary Figure S8. The frequency response of strain for the sensor at different frequencies under the stimulation of a sound pressure level of 90 dB.**

**
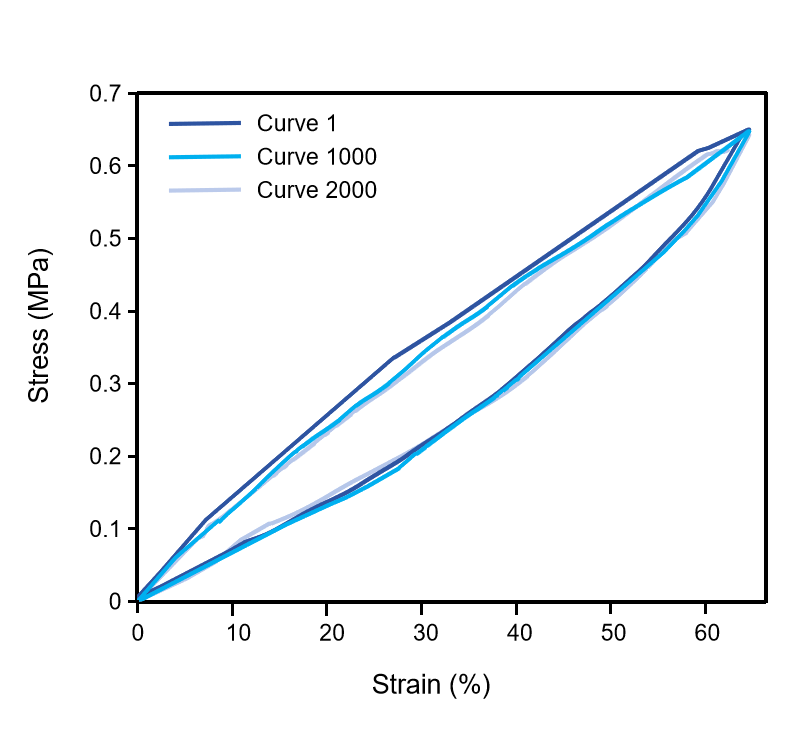
**

**Supplementary Figure S9. Tensile test results of the packaged sensor.** The results indicated that the sensor exhibited no significant mechanical degradation over 2000 stretching cycles.

**
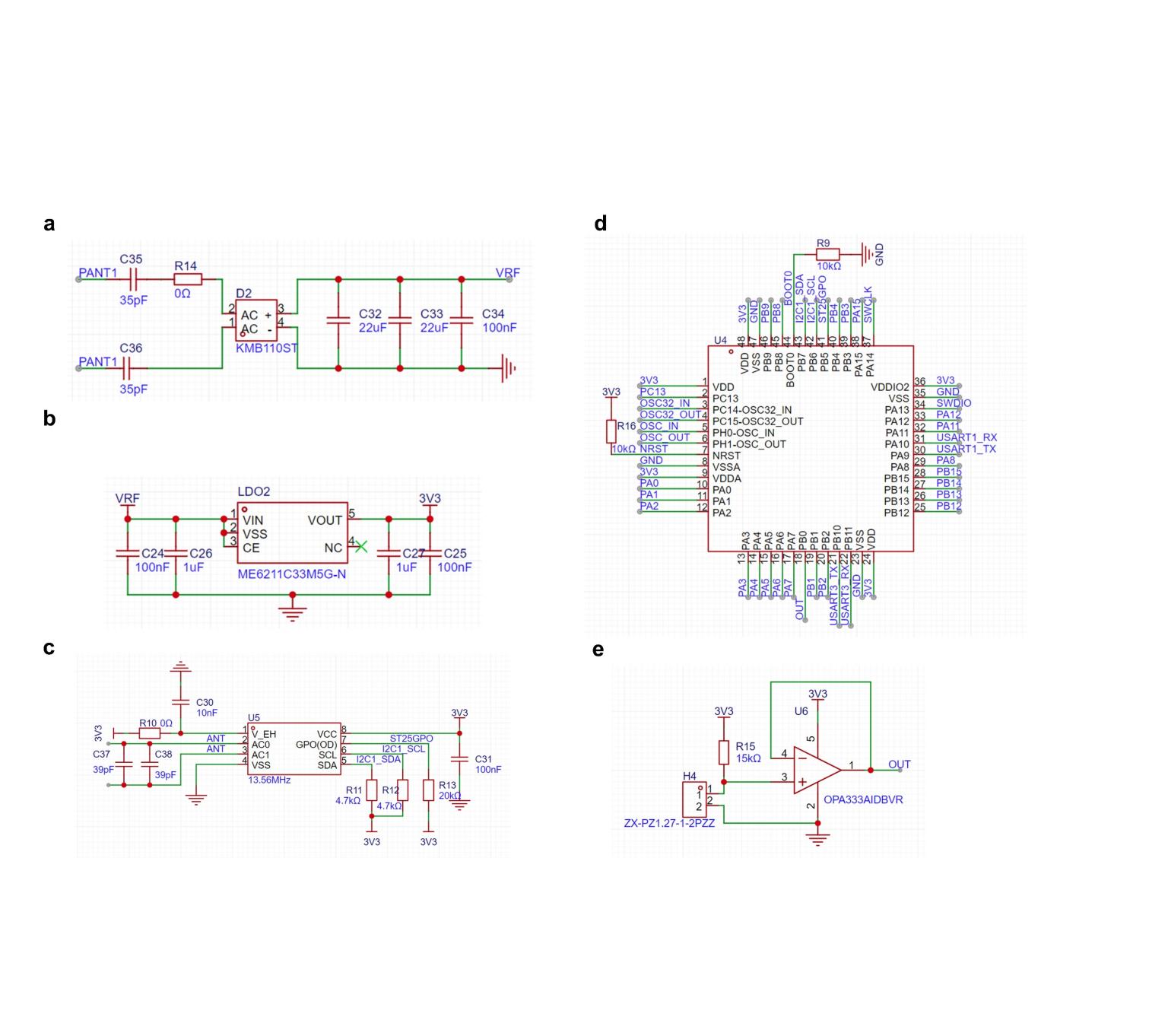
**

**Supplementary Figure S10. Schematic diagram of FPCB.** The circuit design mainly included a) a power management module, b) a voltage regulator circuit, c) an NFC controller, d) a microcontroller circuit based on STM32L051, e) a signal acquisition circuit.


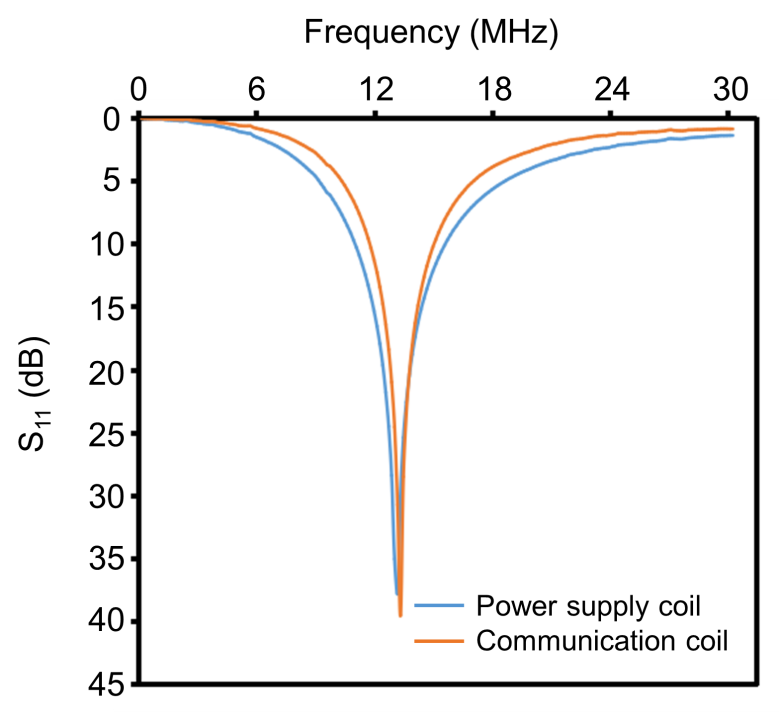


**Supplementary Figure S11. S11-parameters of power coil and communication coil.** The impedances at the resonant frequency were adjusted to ~50 Ω.

**
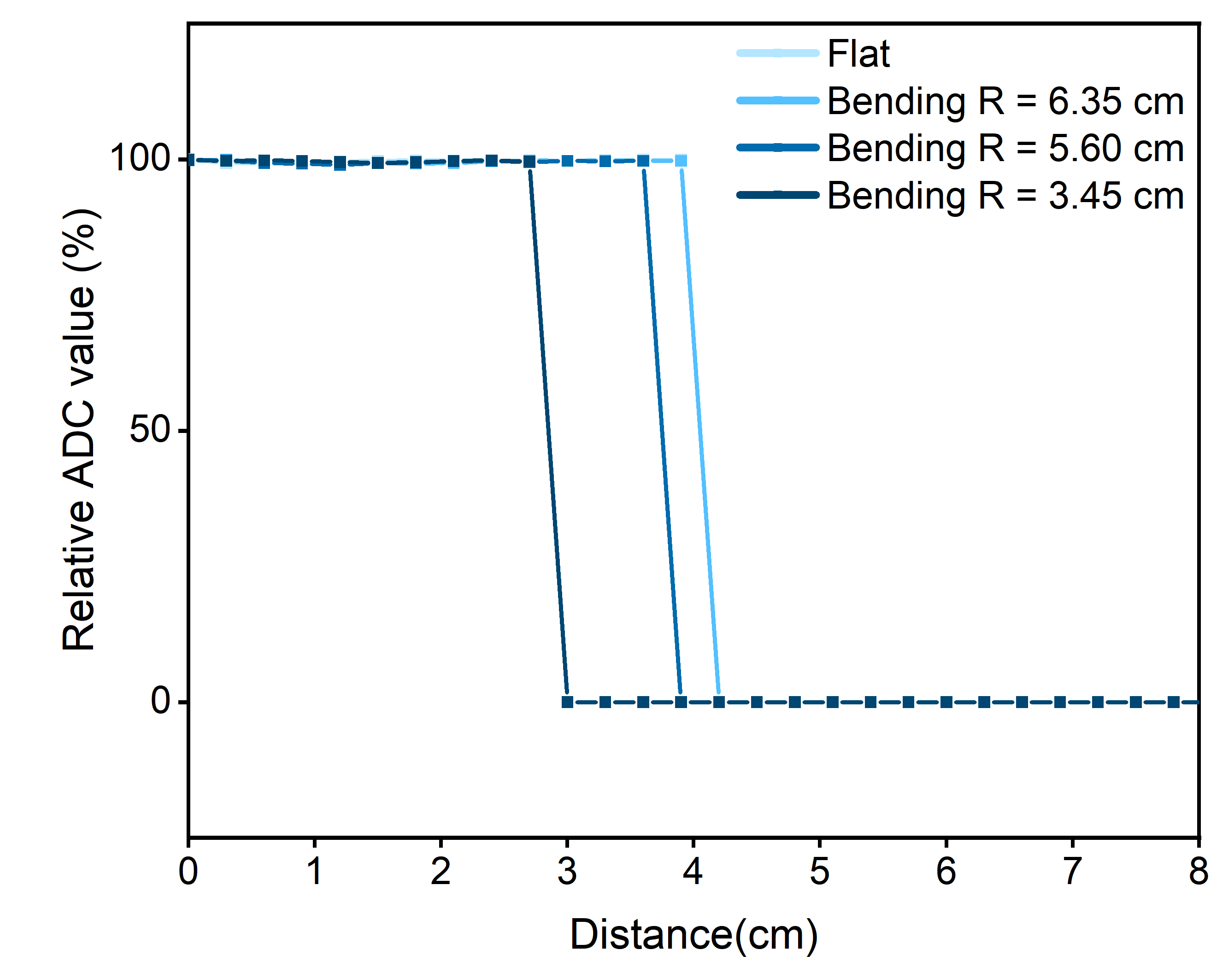
**

**Supplementary Figure S12. Measurement accuracy test of the ATPS.** The ADC value accuracy of ATPS varies with the NFC sensing distance with different bending radii.


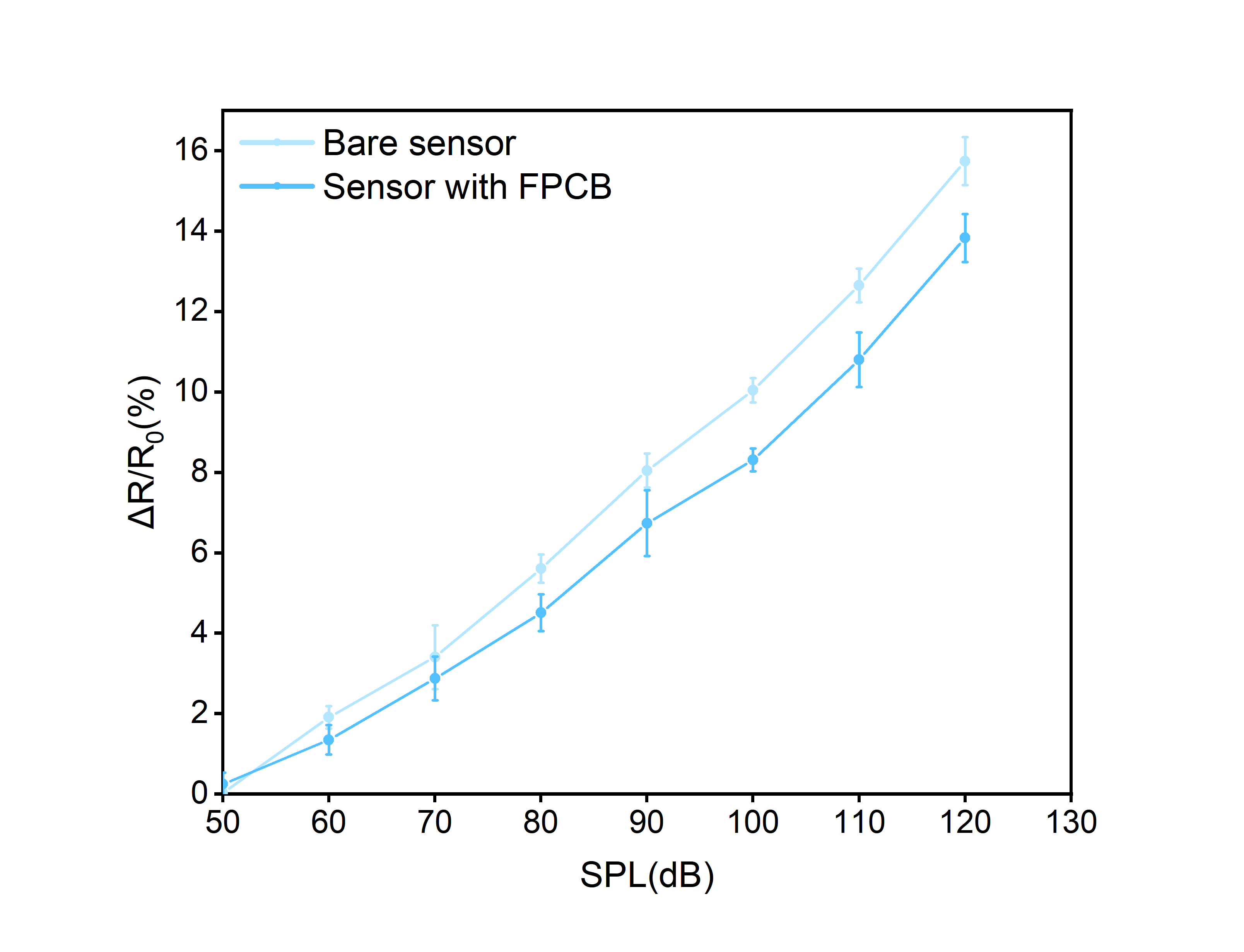


**Supplementary Figure S13. Sensing performance of the sensor with/without FPCB integration.** Comparison of the sensitivity between the bare sensor and the integrated sensor with FPCB at the excitation frequency of 250 Hz.


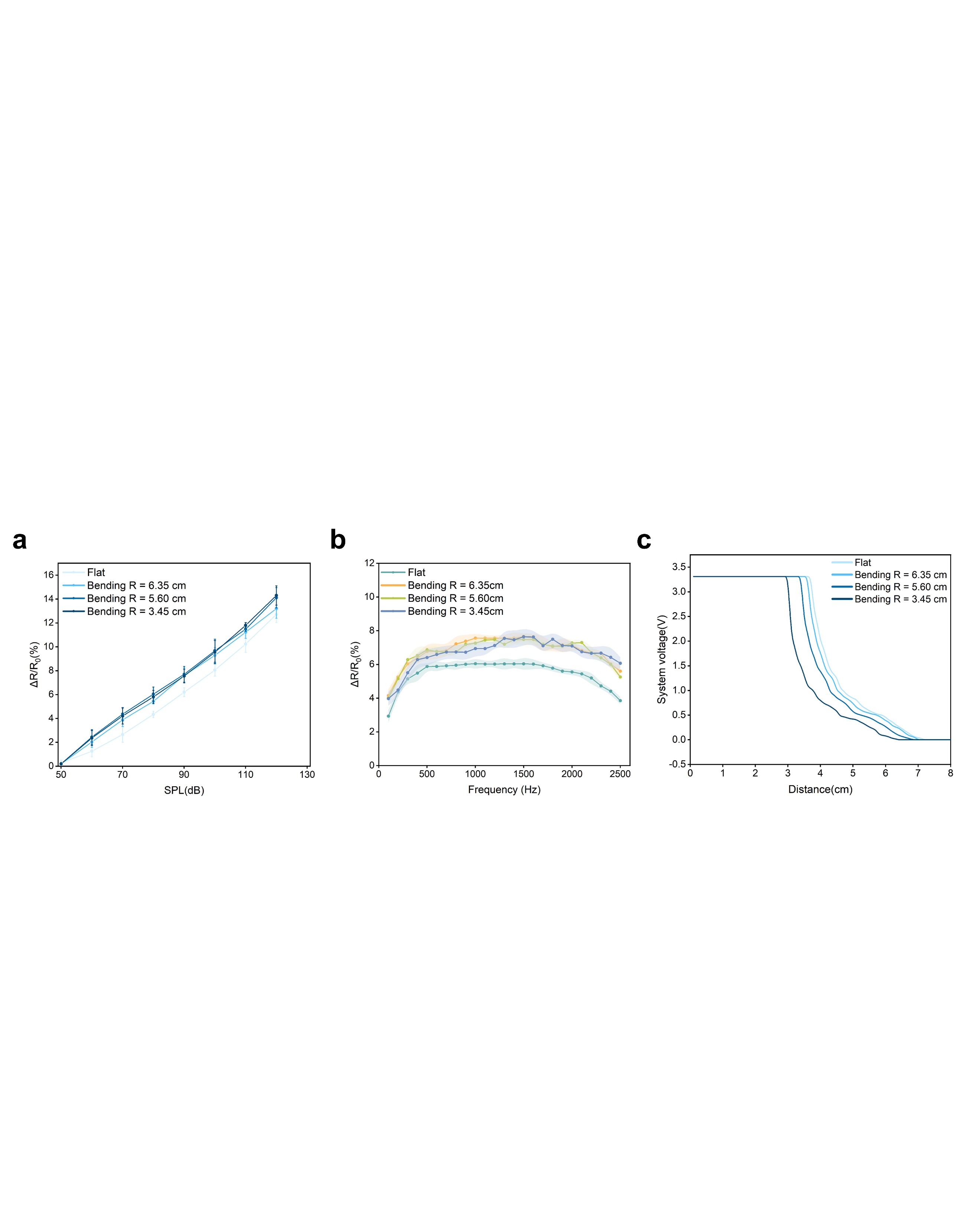


**Supplementary Figure S14. Sensing performance and wireless working distance of the ATPS with different bending deformation.** a) Comparison of sensitivity response under an acoustic excitation of 250 Hz (n = 10). b) Comparison of frequency response under an acoustic excitation of 90 dB (n = 10). c) Comparison of NFC supply voltage change, error bars indicated standard deviation.

**
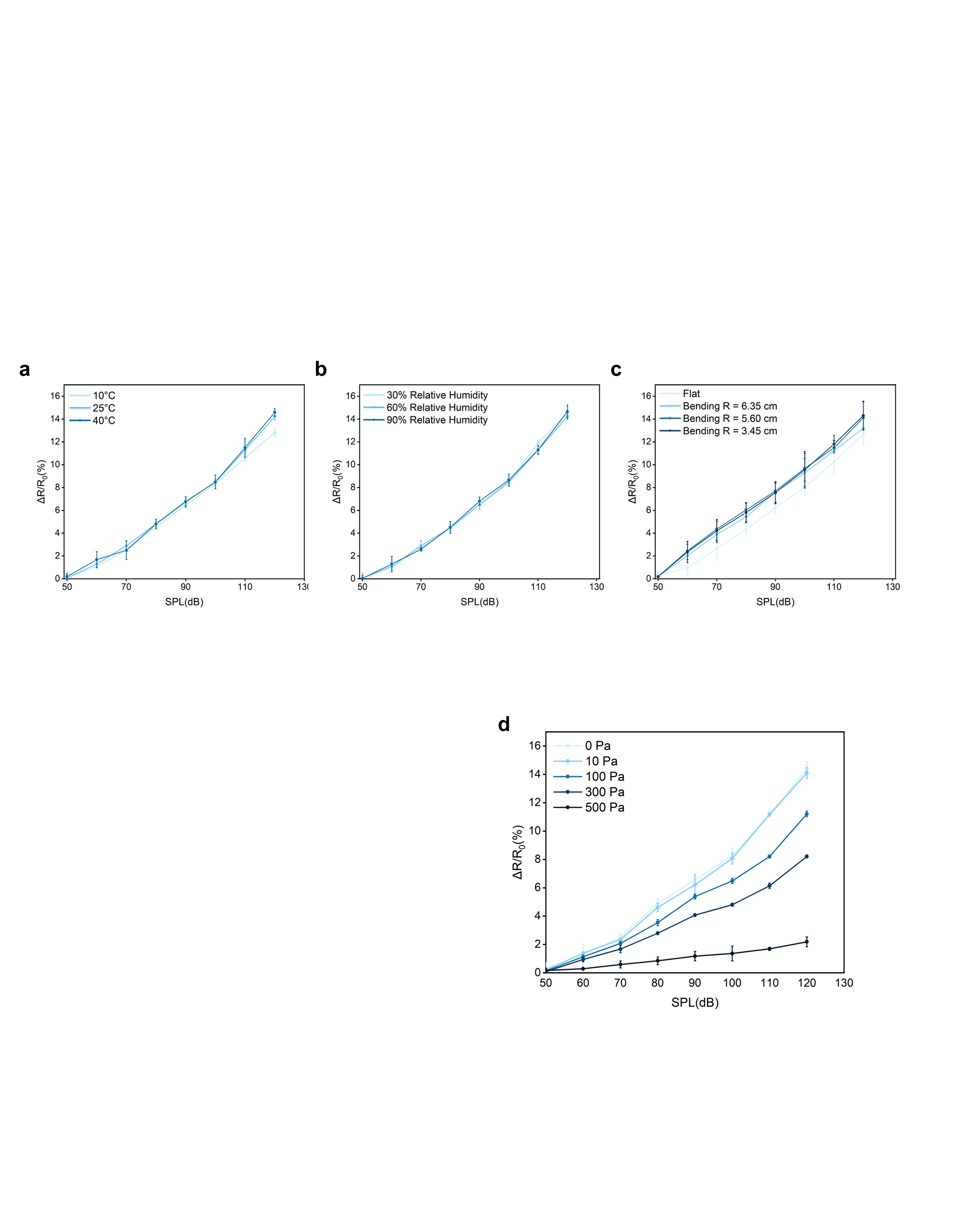
**

**Supplementary Figure S15. Sensitivity tests in different environments.** Intensity response of the integrated sensor with different environmental conditions, including various temperatures (a) and relative humidity (b), n =10, error bars indicated standard deviation.

**
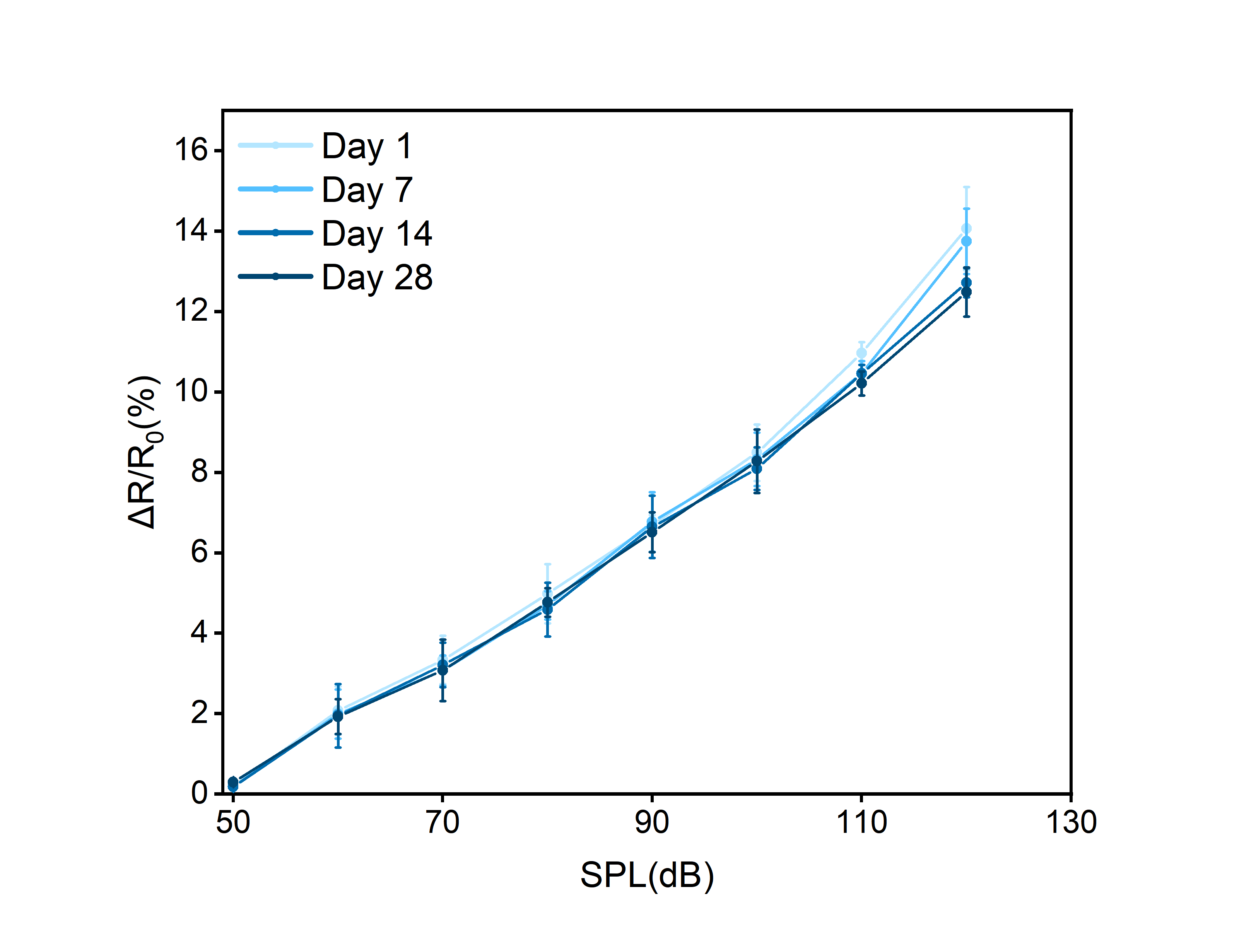
**

**Supplementary Figure S16. Long-term evaluation of the sensor.** The sensitivity of the sensor was tracked in 4 weeks at room temperature, n = 10, error bars indicated the standard deviation.

**
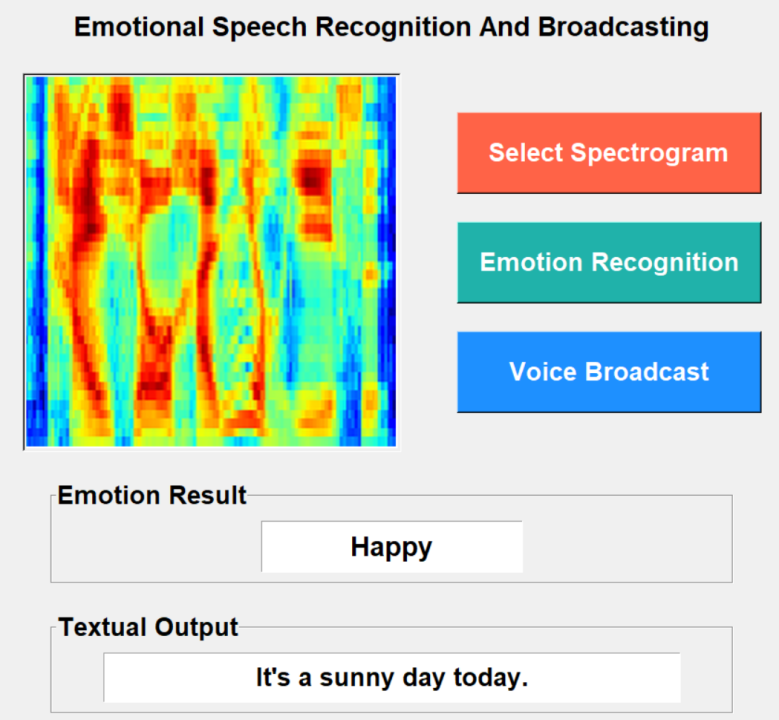
**

**Supplementary Figure S17.** **Graphical user interface (GUI) for emotion and text recognition.**

**
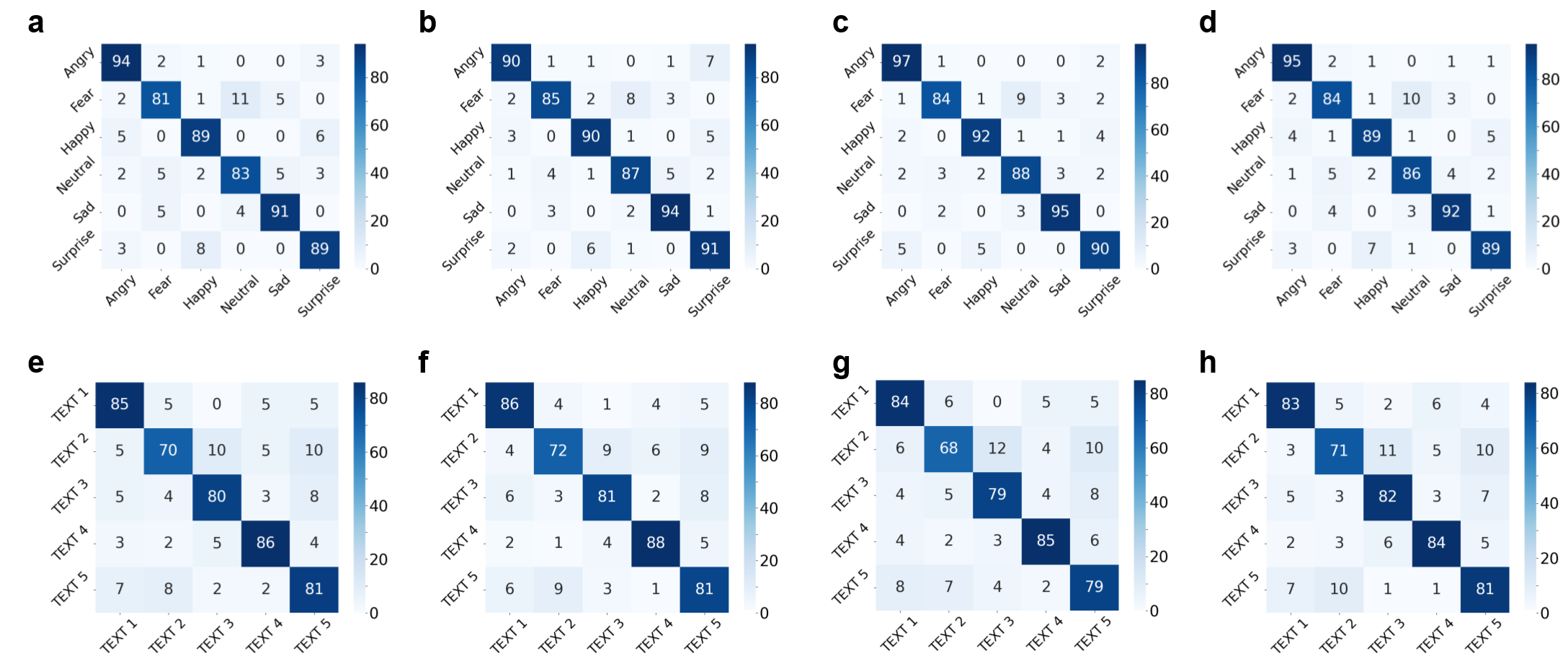
**

**Supplementary Figure S18. Confusion matrices obtained from cross-subject validation experiments.** Average accuracy rate of emotion classification was 87.83%, 89.50%, 91.00% and 89.17%, respectively (a-d). Average accuracy rate of text classification was 80.4%, 81.6%, 79.4% and 80.2% (e-h).

**
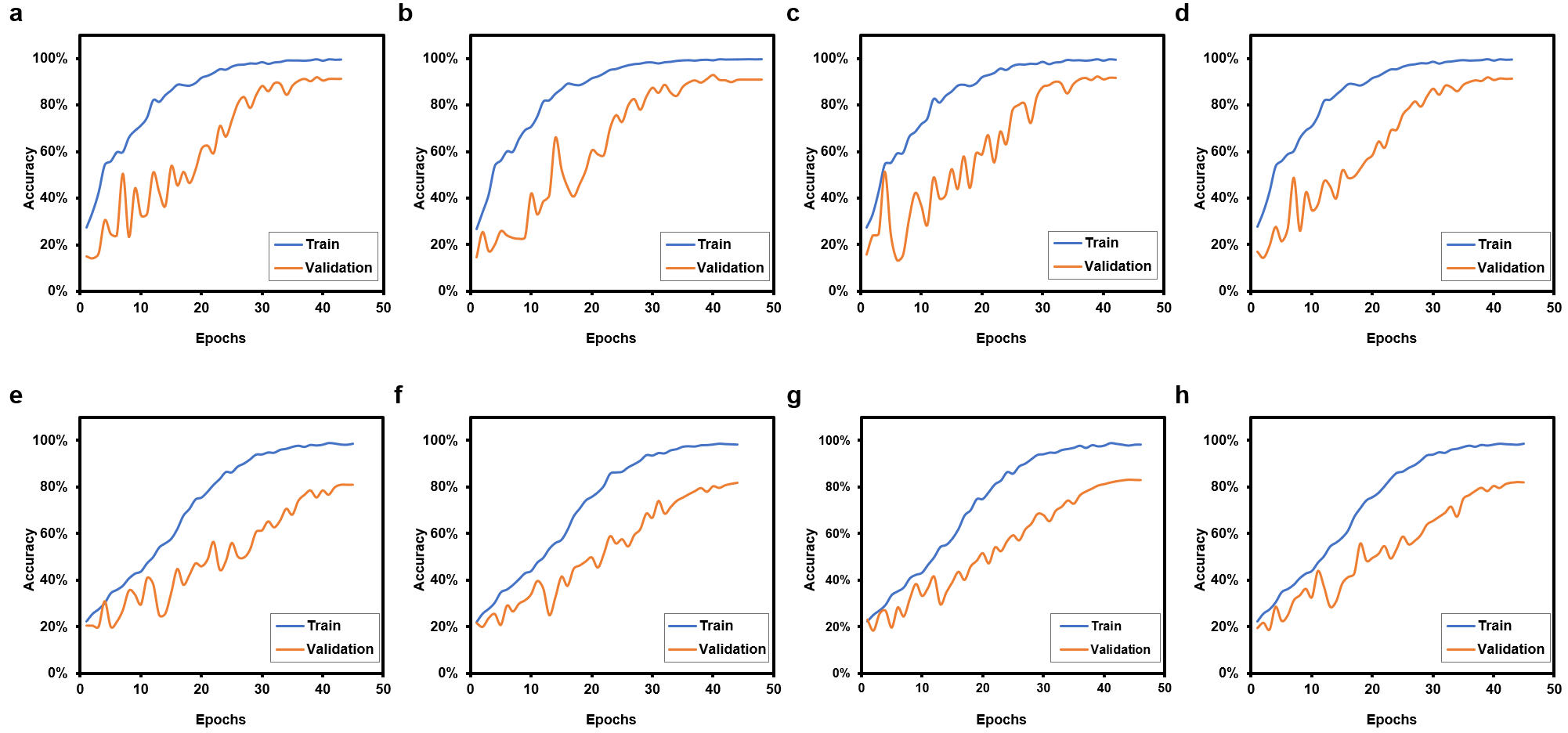
**

**Supplementary Figure S19. The loss curves in the cross-validation experiment.** The validation loss of emotion classification model (a-d) and text classification model (e-h) plateaued without a subsequent increase, and the validation accuracy stabilized closely with the training accuracy.

**
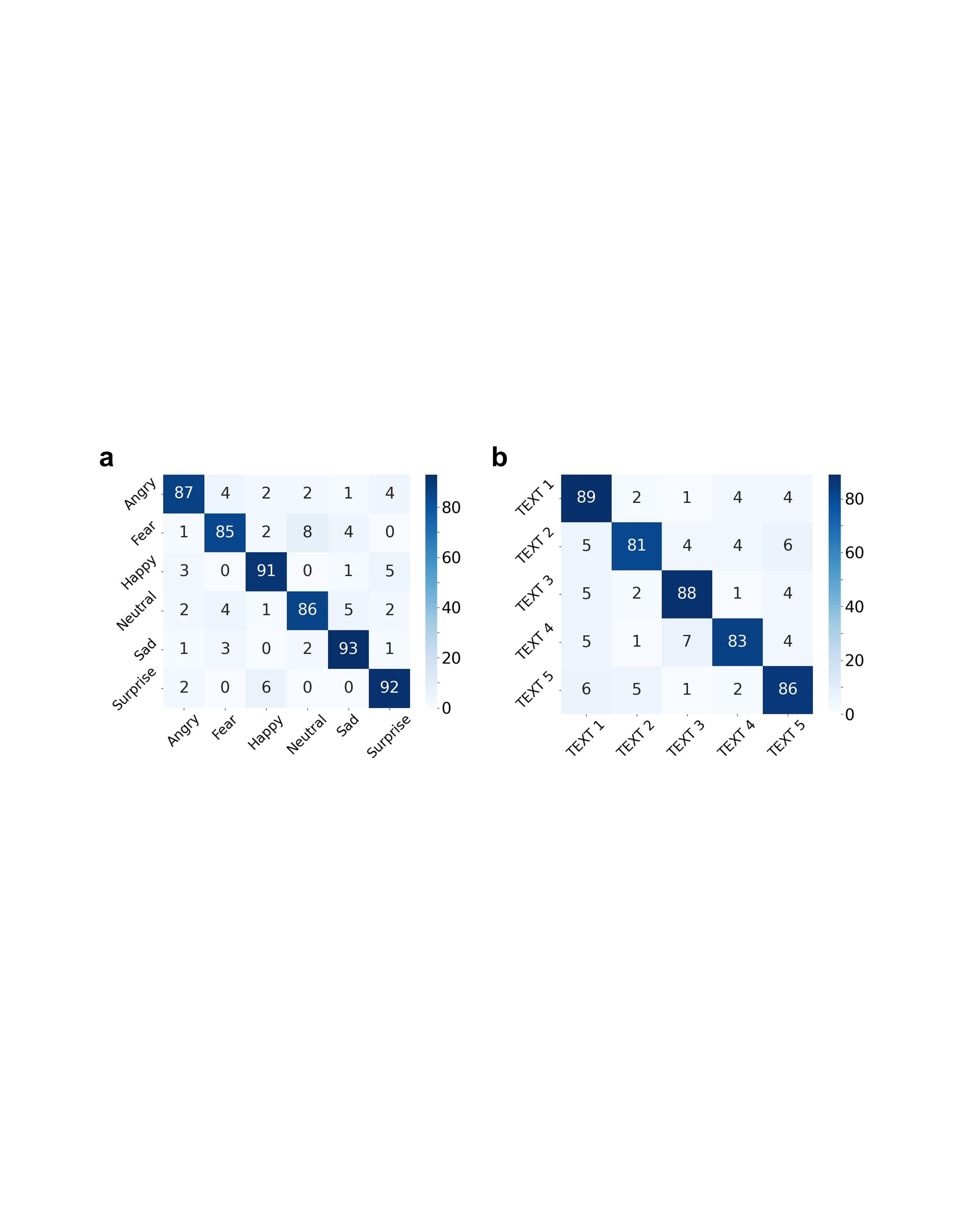
**

**Supplementary Figure S20. Comparison of confusion matrices for the emotion and text recognition tasks using the finalized model on the independent test cohorts.** a, b) The confusion matrix for the (a) emotion recognition results and (b) text recognition on the test sets from an independent subject.

**
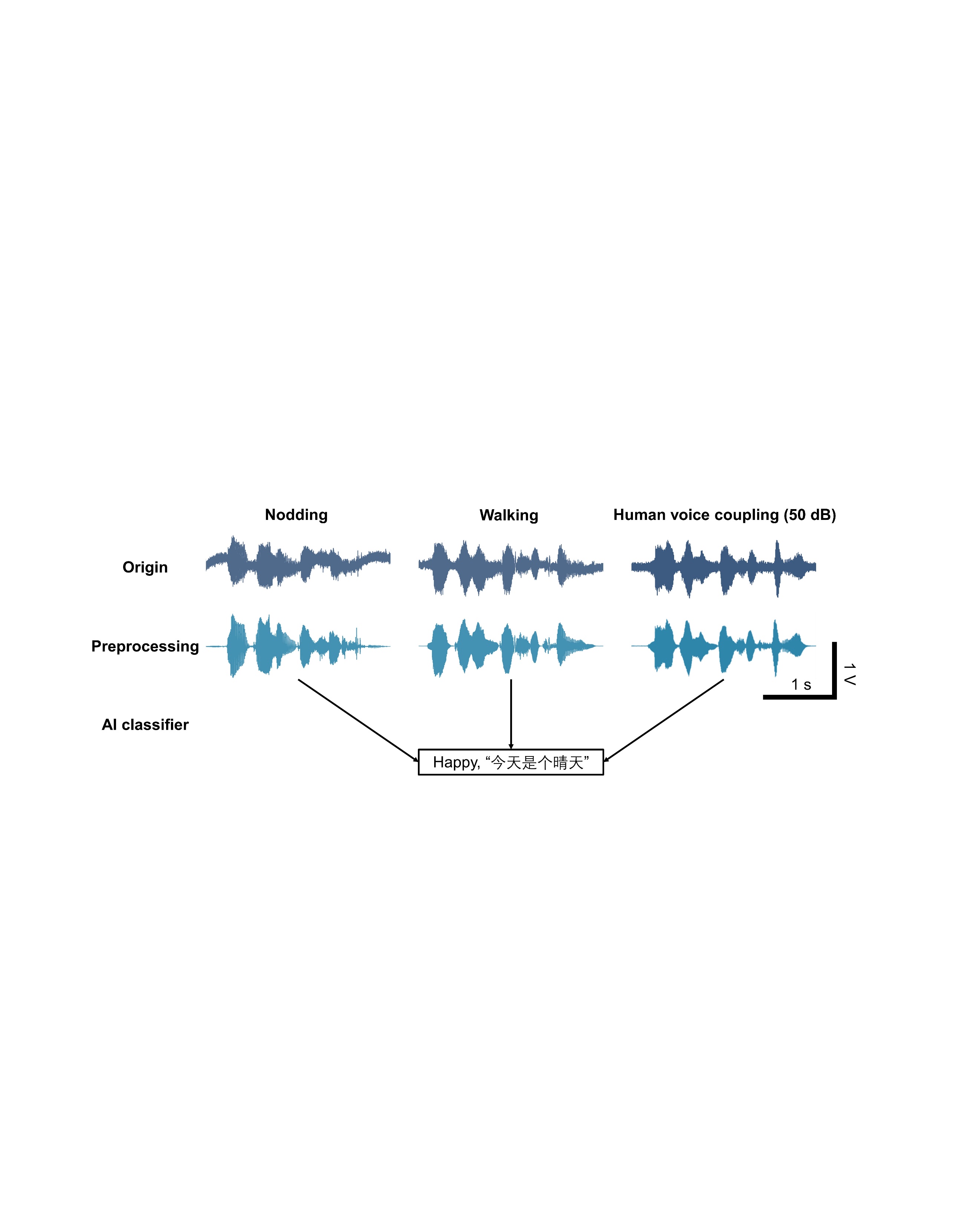
**

**Supplementary Figures S21. Anti-interference capability tests of the ATPS.**

**
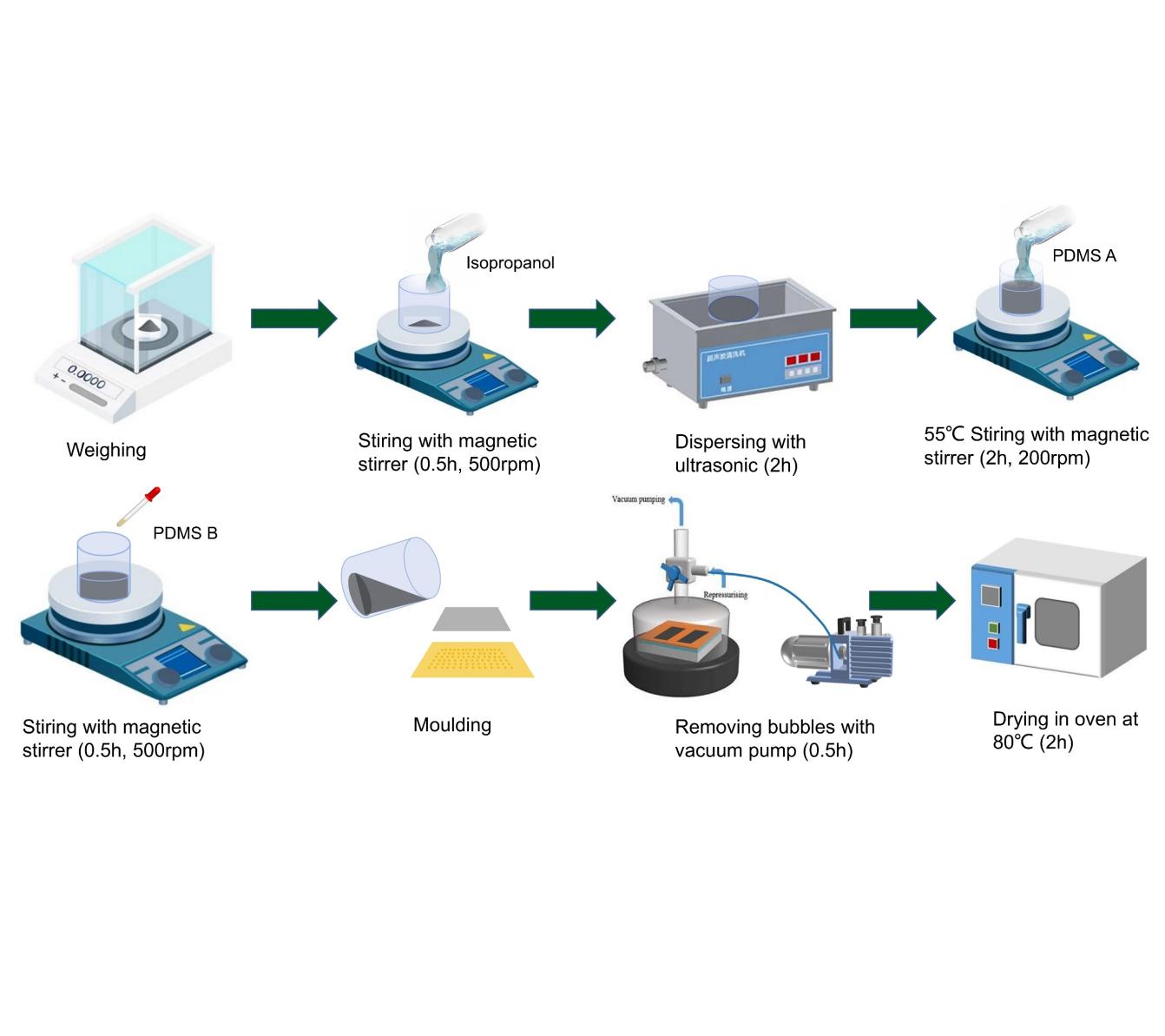
**

**Supplementary Figure S22. Fabrication process of modified MWCNTs and preparation of CNT-PDMS composite films.** Modified MWCNTs were gradually added into PDMS prepolymer at varying weight fractions (0, 2.5, 5, and 7.5 wt%) and thoroughly mixed. After adding the curing agent, the mixture was further stirred and degassed under vacuum to obtain a uniform CNT-PDMS slurry. The slurry was then cast onto either flat silicon wafers or microstructured molds via spin coating. Following a second vacuum degassing step, the films were thermally cured at 80°C for 2 hours to obtain solidified CNT-PDMS composite films, which were subsequently used for multilayer structural encapsulation.

**
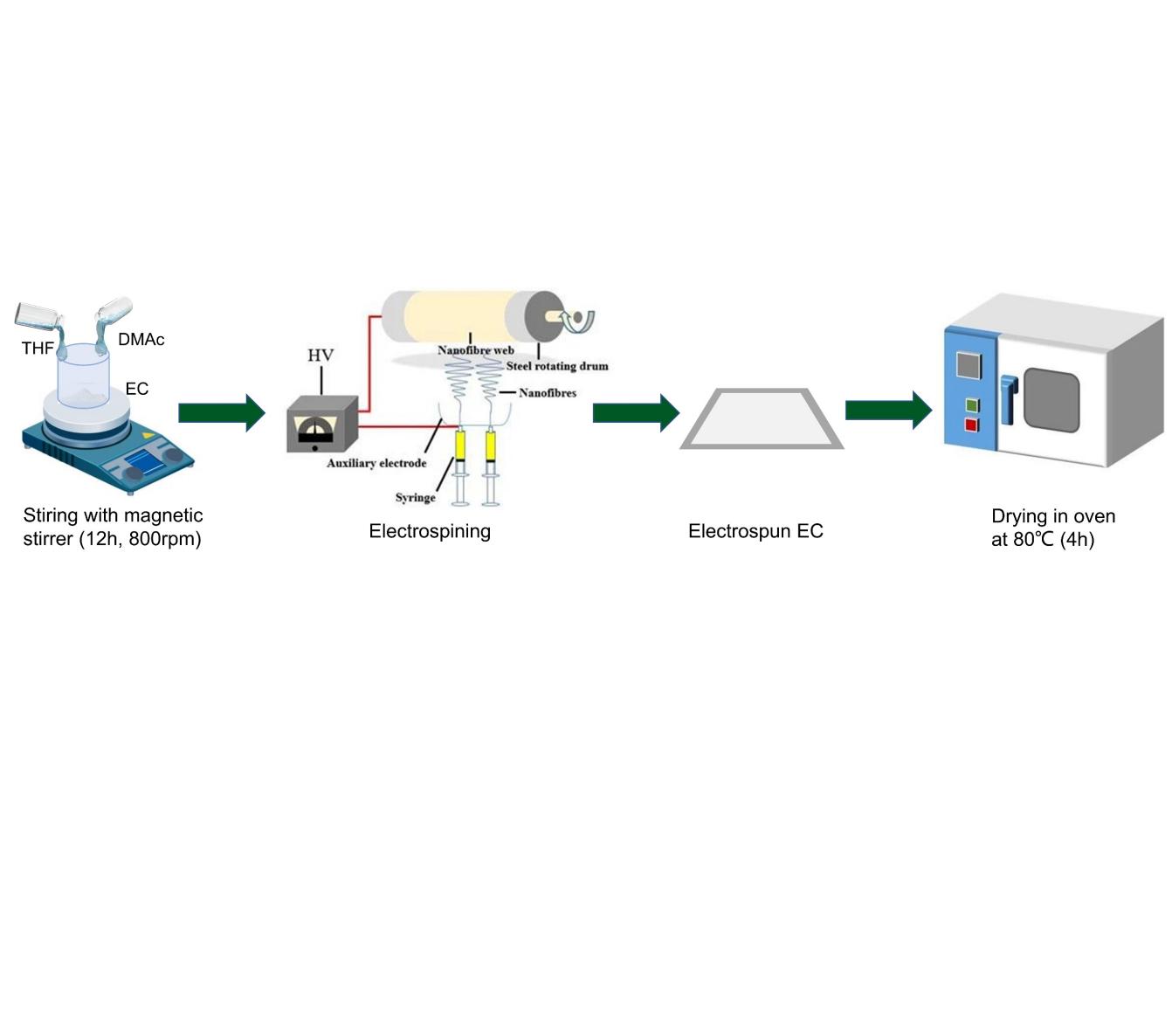
**

**Supplementary Figure S23.** **Fabrication process of electrospun ethyl cellulose (EC) nanofiber membranes.** Tetrahydrofuran (THF) and N, N-dimethylacetamide (DMAc) were mixed at a 1:1 weight ratio, followed by the addition of ethyl cellulose powder to prepare a 10 wt% homogeneous solution. The mixture was stirred magnetically at room temperature for 12 hours to obtain a transparent spinning solution. After defoaming, the solution was loaded into a syringe and connected to an electrospinning apparatus (YLKY-2020A). Electrospinning was conducted continuously for 4 hours under preset parameters. The electrospun EC nanofiber membranes collected on aluminum foil substrates were then removed and dried at 80℃ for 4 hours to eliminate residual solvents. After cooling, the membranes were carefully peeled off and stored in sealed containers for future use.

**
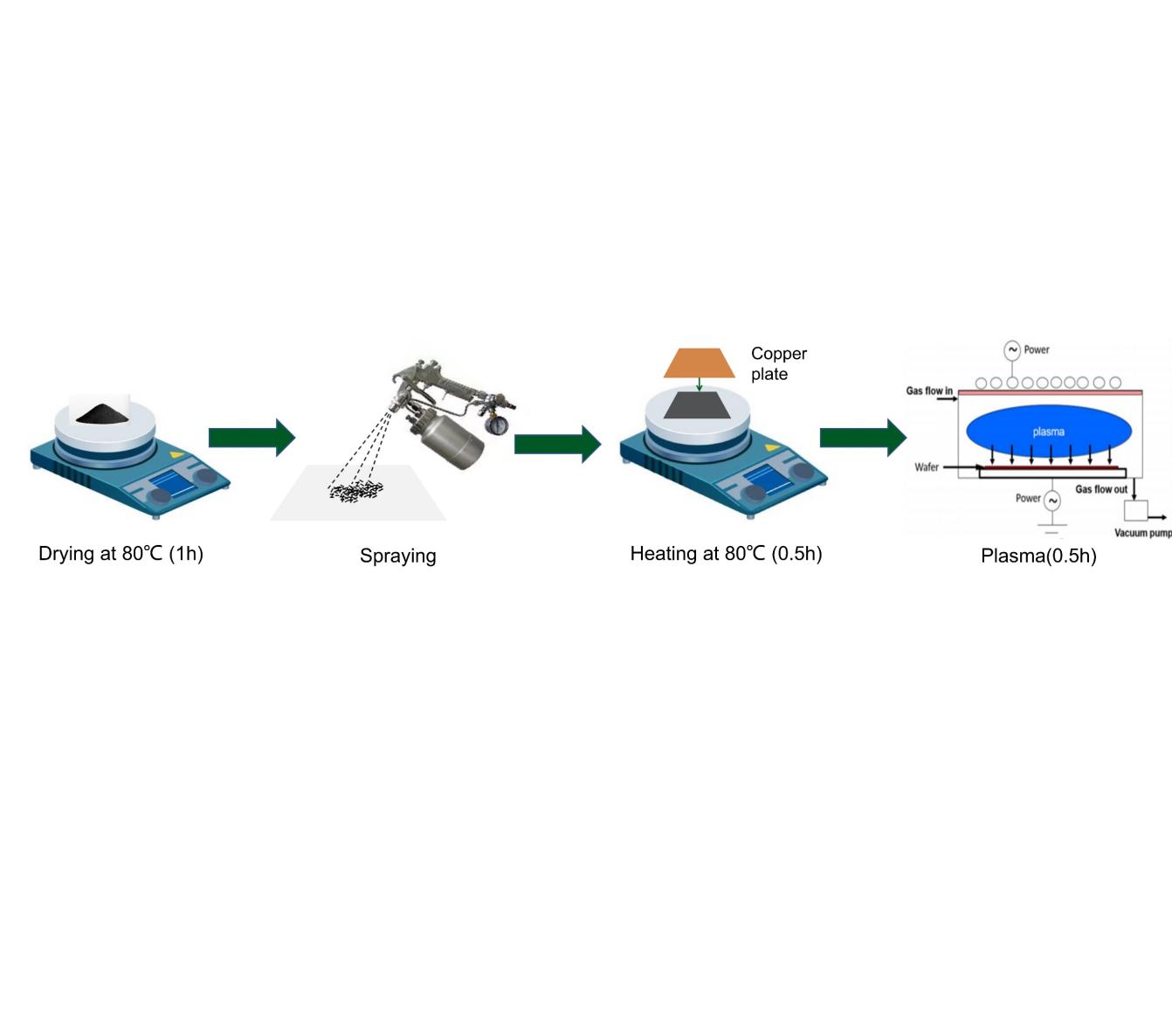
**

**Supplementary Figure S24.** **Fabrication process of the MWCNTs coating layer.** Modified MWCNTs were loaded into a spray bottle and bidirectionally sprayed along the X and Y axes onto the electrospun EC nanofiber membrane to form a uniform MWCNTs coating. The coated membrane was then subjected to thermal treatment at 80 °C and compacted under 5 kPa to optimize the conductive network. Subsequently, plasma surface activation was applied to enhance the interfacial adhesion between the MWCNTs and the fiber membrane, resulting in a stable, uniform, and peel-resistant conductive composite film.

**
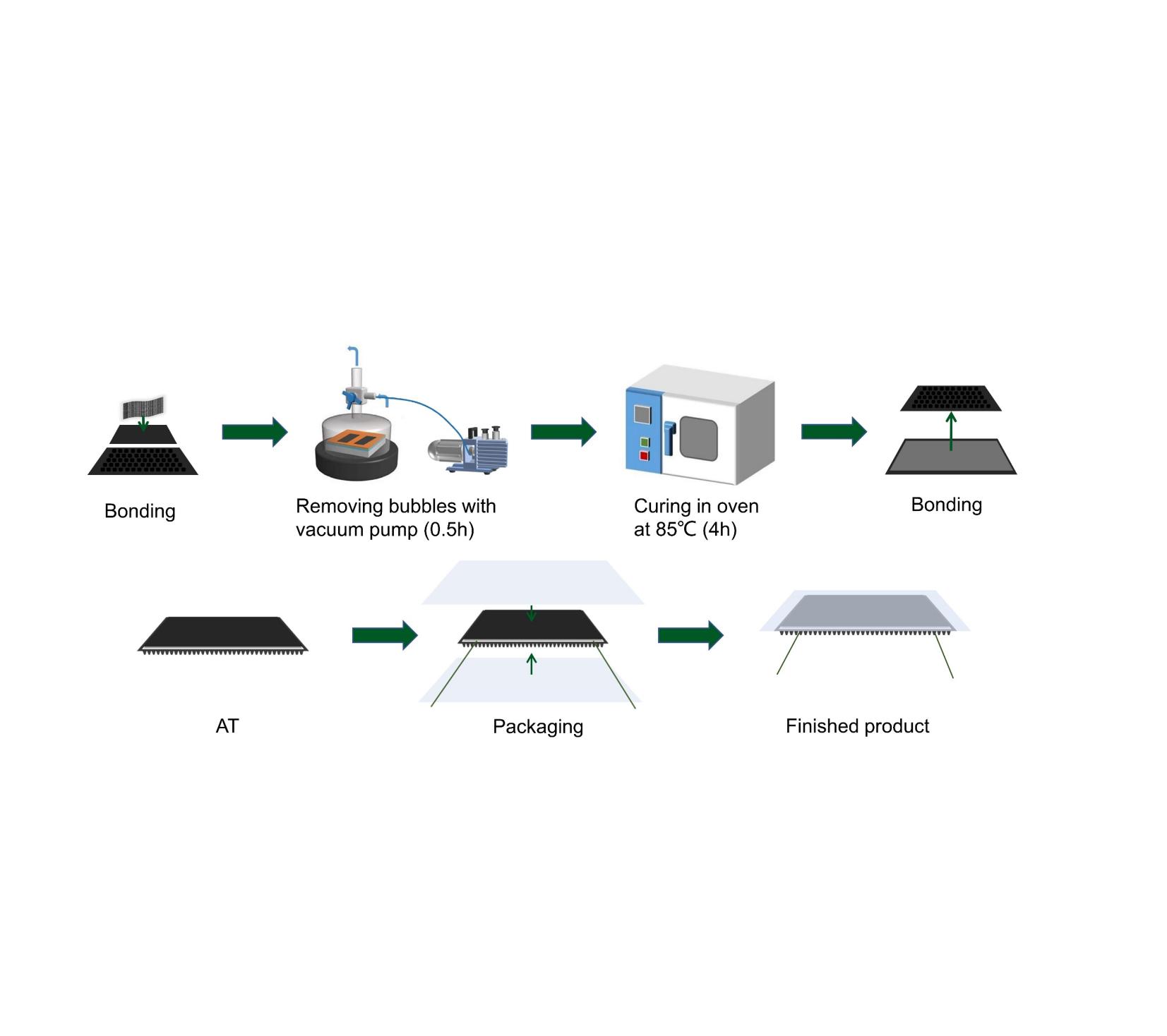
**

**Supplementary Figure S25.** **Lamination and encapsulation process of multilayer films for device integration.** The MWCNTs-coated electrospun EC nanofiber membrane was first aligned and laminated onto the surface of the first-layer CNT-PDMS film. After vacuum degassing, the assembly was dried and cured at 85°C to form a composite layer. This composite was then bonded to a microstructured CNT-PDMS layer containing conical micro-pillars via plasma activation and pressure-assisted curing. Conductive silver paste was used to attach copper wires for electrical connection. Finally, a PU protective film with an open window over the sensing region was applied, and the entire device was affixed to the laryngeal area of the neck for signal detection.

**
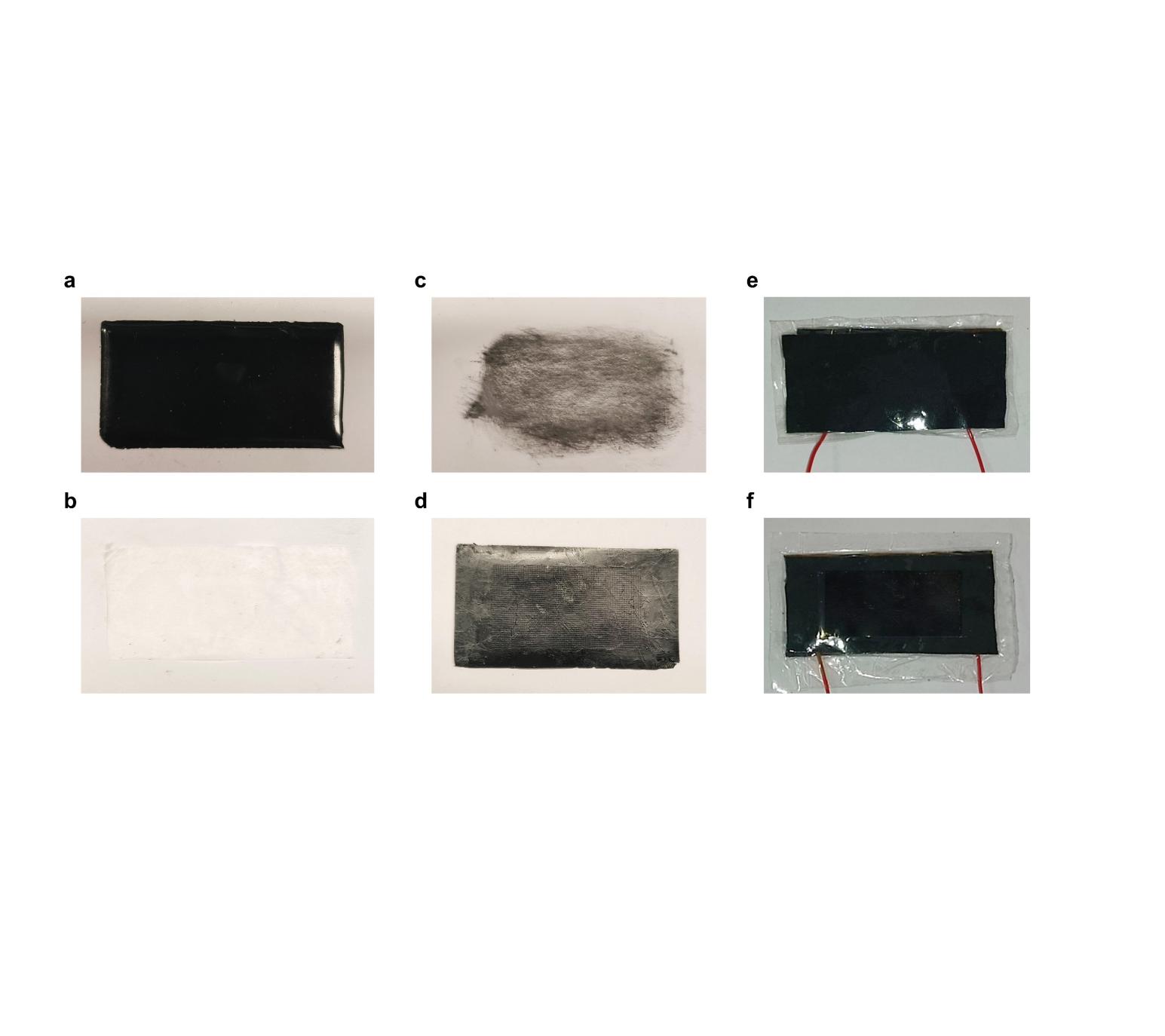
**

**Supplementary Figure S26.** **Photographs of the sensor.** a-d) Exploded view of the sensors structure, showing the individual layers in sequence: (a) CNT-PDMS substrate, (b) electrospun EC nanofiber, (c) MWCNTs conductive coating, and (d) CNT-PDMS layer with microstructure. e, f) Front (e) and back (f) views of the packaged sensor.

**Supplementary Notes**

**Supplementary Note S1. Mechanical stimulation and response analysis of the ATPS.**

To assess the mechanical sensitivity of the artificial throat patch system (ATPS), a calibrated microphone was used to deliver controlled acoustic stimuli to mimic throat vibrations near the sensor surface.

1) Estimation of Vocal Vibration-Induced Force

Typical vocal fold vibration occurs at 100 - 2500 Hz with amplitudes of 0.1 - 1 mm. The generated force was approximated as:

$\text{F}\text{ }\text{=}\text{ }\text{ma}\text{ }\text{=}\text{ }\text{m}{\text{(2π}\text{f}\text{ }\text{)}}^{\text{2}}\text{A}$ (1)

Using minimal parameters (*m* = 0.5 g, *f* = 100 Hz, *A* = 0.1 mm), the force was ~ 0.02 N, while intense vocal vibration could approach ~1 N.

2) Estimation of Force Induced by Acoustic Excitation

Sound pressure P from the speaker was estimated as:

$\text{P =}\text{ P}_{\text{0}}\text{×}\text{10}^{\frac{SPL}{\text{20}}}$ (2)

where $\text{P}_{\text{0}}$ = 20 μPa was the reference sound pressure in air.

At 60 -130 dB, *P* ranged from 0.02 to 63.2 Pa.

With an ATPS sensing area of 4 × 2 cm², the corresponding force was 16 μN to 0.506 N.

3) Validity of Acoustic Stimulation and Sensor Responsiveness

The acoustic force range overlaped with throat-generated forces, confirming the physical relevance of the method. Despite transmission losses, the ATPS consistently exhibited measurable resistance changes, verifying its high sensitivity and the feasibility of using acoustic excitation as a reproducible evaluation approach.

**Supplementary Note S2.** **Signal preprocessing procedure.**

To improve signal quality and ensure reliable feature extraction, the raw voltage signals from the ATPS were processed through the following steps:

1) Denoising and Smoothing

A 4th-order Butterworth bandpass filter (50-4000 Hz) was applied to remove baseline drift and high-frequency noise. A Savitzky-Golay filter (window length = 11, order = 2) was used to smooth the signal.

2) Normalization

Z-score normalization was performed to standardize the signal to zero mean and unit variance, reducing variability across subjects and recordings.

3) Pre-emphasis

To compensate for the natural attenuation of high-frequency components introduced by the vocal tract and sensor structure, a first-order high-pass pre-emphasis filter was applied as:

$\text{y}\text{(}\text{n}\text{)}\text{ }\text{=}\text{ }\text{x}\text{(}\text{n}\text{)-0.98}\text{x}\text{(}\text{n}\text{-1)}$ (3)

where the pre-emphasis coefficient α was set to 0.98.

4) Framing and Windowing
The pre-emphasized signal was divided into overlapping frames of 200 samples (50 ms), with a frame shift of 100 samples (25 ms). Each frame was multiplied by a Hamming window to reduce spectral leakage. The window function is defined as:

*S*$\text{(}\text{n}\text{)}\text{=}\left\{ \begin{aligned} \text{0.54-}\text{β}\text{cos(}\frac{\text{2π}\text{n}}{\text{N}\text{-1}}\text{), }\text{0}\text{ }\text{≤}\text{ }\text{n}\text{ }\text{≤}\text{ }\text{N}\text{-1} \\ \text{0}\text{ }\text{, }\text{others} \end{aligned} \right.$ (4)

where *N* = 200 and the window coefficient *β* = 0.46.

5) Endpoint Detection

A double-threshold algorithm based on short-term energy and zero-crossing rate was used to detect voiced segments and remove silence.

**Supplementary Note S3. Feature extraction procedure.**

To extract meaningful acoustic features from the preprocessed throat vibration signals, Mel-Frequency Cepstral Coefficients (MFCCs) were computed as follows:

1) Fast Fourier Transform (FFT)

For the *i*-th frame $x_{i}$(*n*), *n* = 0, 1,…, *N*−1, *N* = 200, the FFT was computed to obtain the frequency domain representation:

$\text{F}_{\text{i}}\text{(}\text{k}\text{)}\text{ }\text{=}\text{ }\sum_{\text{n}\text{=0}}^{\text{N}\text{-}\text{1}} \text{x}_{\text{i}}\text{(}\text{n}\text{)}\text{e}^{\frac{\text{-}\text{j}\text{2}\text{π}}{\text{N}}\text{kn}}$ (5)

The power spectrum was then calculated as:

$\text{P}_{\text{i}}\text{(}\text{k}\text{)}\text{ }\text{= }\frac{{\text{|}\text{F}_{\text{i}}\text{(}\text{k}\text{)|}}^{\text{2}}}{\text{N}}$ (6)

2) Mel Filter Bank Processing

The power spectrum was passed through a 40-channel Mel filter bank to simulate the human auditory scale. Filter spacing increased with frequency, and each filter output represented perceptual band energy. The Mel-filtered energy for each frame was computed as:

$\text{H}_{\text{m}}\text{(}\text{k}\text{)}\text{ }\text{=}\text{ }\left\{ \begin{matrix} \text{0} & \text{k}\text{ }\text{<}\text{ }\text{f}\text{(}\text{m}\text{-}\text{1}\text{)} \\ \frac{\text{k}\text{-}\text{f}\text{(}\text{m}\text{-}\text{1}\text{)}}{\text{f}\text{(}\text{m}\text{)-}\text{f}\text{(}\text{m}\text{-}\text{1}\text{)}} & \text{f}\text{(}\text{m}\text{-}\text{1}\text{)}\text{ }\text{≤}\text{ }\text{k}\text{ }\text{<}\text{ }\text{f}\text{(}\text{m}\text{)} \\ \text{1} & \text{k}\text{ }\text{=}\text{ }\text{f}\text{(}\text{m}\text{)} \\ \frac{\text{f}\text{(}\text{m}\text{+}\text{1}\text{)-}\text{k}}{\text{f}\text{(}\text{m}\text{+}\text{1}\text{)-}\text{f}\text{(}\text{m}\text{)}} & \text{f}\text{(}\text{m}\text{)}\text{ }\text{<}\text{ }\text{k}\text{ }\text{≤}\text{ }\text{f}\text{(}\text{m}\text{+}\text{1}\text{)} \\ \text{0} & \text{k}\text{ }\text{>}\text{ }\text{f}\text{(}\text{m}\text{+}\text{1}\text{)} \end{matrix} \right.$ (7)

$\text{M}_{\text{i}}\text{(}\text{m}\text{) =}\text{ }\sum_{\text{k}} \text{P}_{\text{i}}\text{(}\text{k}\text{)×}\text{H}_{\text{m}}\text{(}\text{k}\text{), }\text{m}\text{ = 1, 2, ..., 40}$ (8)

where $\text{H}_{\text{m}}\text{(}\text{k}\text{)}$ denotes the weighting function of the *m*-th filter at frequency bin *k*. The filters are evenly spaced on the Mel scale, simulating the frequency sensitivity of human auditory system.

3) Logarithmic Compression and Discrete Cosine Transform (DCT)

The logarithm of Mel filter outputs $\log\text{M}_{\text{i}}\text{(}\text{m}\text{)}$ was taken, followed by DCT to yield cepstral coefficients:

*C*$\text{(}\text{u}_{\text{i}}\text{) =}\sum_{\text{m}\text{=1}}^{\text{40}} \log\text{M}_{\text{i}}\text{(}\text{m}\text{)}\text{cos(}\frac{\text{(}\text{m}\text{-0.5)}\text{u}\text{π}}{\text{40}}\text{), }\text{u}\text{ = 0,1,...,13}$ (9)

The first 13 coefficients were retained as static MFCC features, representing the spectral envelope.

4) Dynamic Feature Computation
To capture temporal dynamics, first and second-order delta coefficients were computed to reflect the velocity and acceleration of spectral changes:

${\text{∆}\text{C}}_{\text{i}}\text{(}\text{u}\text{)}\text{ }\text{=}\text{ }\frac{\text{C}_{\text{i+d}}\text{(}\text{u}\text{)-}\text{C}_{\text{i-d}}\text{(}\text{u}\text{)}}{\text{2}\text{d}}$ (10)

${\text{∆}^{\text{2}}\text{C}}_{\text{i}}\text{(}\text{u}\text{)}\text{ }\text{=}\text{ }\frac{{\text{∆}\text{C}}_{\text{i+d}}\text{(}\text{u}\text{)-}{\text{∆}\text{C}}_{\text{i-d}}\text{(}\text{u}\text{)}}{\text{2}\text{d}}$ (11)

where the delta window *d* was set to 1.

5) Feature Integration

Static MFCCs, along with their first and second-order derivatives and log frame energy, were concatenated to form a 40-dimensional feature vector per frame. With 128 frames per signal, the final feature matrix dimension was 40×128, serving as input to the subsequent emotion recognition model.

**Supplementary Note S4. Hybrid deep learning architecture for emotion recognition.**

To effectively capture emotional cues from throat vibration-based speech signals, a hybrid model combining CNN, Bi-LSTM, and Transformer encoders was designed. This architecture enabled local time-frequency pattern extraction, temporal modeling, and global context aggregation.

1) CNN Module: Local Time-Frequency Feature Extraction

Input: 40×128 MFCC matrix

Conv Layer 1: 3×3 kernel, 32 filters, ReLU + 2×2 MaxPooling

Conv Layer 2: 3×3 kernel, 64 filters, ReLU + MaxPooling

The output was flattened into a temporal sequence (*T* = 32, each step = 640-dim) for sequential processing.

2) Bi-LSTM Module: Bidirectional Temporal Modeling

Two stacked Bi-LSTM layers (128 units each, return_sequences = True)

Dropout (rate = 0.3) between layers

This module captured bidirectional time dependencies crucial for emotion dynamics.

3) Transformer Encoder Module: Global Context Modeling

Two encoder layers with 8-head self-attention (dim = 128), FFN (hidden dim = 512)

Positional encoding added to Bi-LSTM outputs

Each layer included residual connections and layer normalization

This allowed efficient global dependency learning across the entire speech sequence.

4) Output and Classification Layers

GlobalAveragePooling1D → Dense (128, ReLU) → Dropout (0.5) → Dense (6, Softmax)

Output: Six emotion classes (Angry, Fear, Happy, Neutral, Sad, Surprise)

Also adaptable for five-class content classification (e.g., “Let’s go for dinner”, etc.)

**Supplementary Note S5. Model training strategy.**

To ensure robust generalization and stable convergence, the model was trained using the following protocol:

1) Dataset Partitioning

The dataset was divided into training, validation and test sets, of which 900 data points collected from 3 subjects were used as the training set and validation set (8:2), while the 300 data points left (from another subject) were test set to balance model learning and performance evaluation.

2) Loss Function and Optimizer

Categorical Cross-Entropy was used for multi-class classification.

Optimization was performed with Adam (learning rate = 0.001, *β*₁ = 0.9, *β*₂ = 0.999), selected for its adaptive learning and stability.

3) Training Configuration

Batch size: 32

Maximum epochs: 100

Model parameters were updated per epoch using the training set, with validation loss monitored after each epoch.

4) Early Stopping

Training stopped if validation loss did not improve for 10 consecutive epochs. The best-performing model parameters were restored automatically.

**Supplementary References**

1. Su et al. Spider silk-inspired environmentally adaptive intelligent graphene artificial throat. Chemical Engineering Journal, 2025, 511: 162177.
2. Wei Y, Qiao Y, Jiang G, et al. A wearable skinlike ultra-sensitive artificial graphene throat. ACS nano, 2019, 13(8): 8639-8647.
3. Yang et al. Mixed-modality speech recognition and interaction using a wearable artificial throat. Nature Machine Intelligence, 2023, 5(2): 169-180.
4. Faeghifard et al. Fabrication and Investigation on Mechanical, Electrical, and Sensing Performance of Polydimethylsiloxane/Carbon Nanotube and Thermoplastic Polyurethane/Carbon Nanotube Wearable Strain Sensors. Advanced Engineering Materials, 2025, 27(7): 2402208.
5. Wang et al. Flexible and high-performance piezoresistive strain sensors based on multi-walled carbon nanotubes@ polyurethane foam. RSC advances, 2022, 12(22): 14190-14196.
6. Wu et al. Piezoresistive stretchable strain sensors with human machine interface demonstrations. Sensors and Actuators A: Physical, 2018, 279: 46-52.
7. Li et al. Anisotropic structural carbon nanotube aerogels for piezoresistive strain sensors with multidirectional sensitivity. Composites Part B: Engineering, 2025, 291: 112028.
8. Su et al. Spider silk-inspired environmentally adaptive intelligent graphene artificial throat. Chemical Engineering Journal, 2025, 511: 162177.
9. Wei Y, Qiao Y, Jiang G, et al. A wearable skinlike ultra-sensitive artificial graphene throat. ACS nano, 2019, 13(8): 8639-8647.
10. Yang et al. Mixed-modality speech recognition and interaction using a wearable artificial throat. Nature Machine Intelligence, 2023, 5(2): 169-180.
11. Mao et al. Machine Learning-Enabled Emotion Recognition by Multisource Throat Signals. ACS nano, 2025, 19(19): 18397-18408.
12. Wang et al. All-weather, natural silent speech recognition via machine-learning-assisted tattoo-like electronics. npj Flexible Electronics, 2021, 5(1): 20.
13. He et al. Portable and Self-Powered Sensing AI-Enabled Mask for Emotional Recognition in Virtual Reality. ACS Applied Materials & Interfaces, 2025, 17(12): 19175-19188.
14. Lee et al. Encoding of multi-modal emotional information via personalized skin-integrated wireless facial interface. Nature Communications. 2024, 15(1): 530.
15. Yang et al. Adhesive and hydrophobic bilayer hydrogel enabled on‐skin biosensors for high‐fidelity classification of human emotion. Advanced Functional Materials, 2022, 32(29): 2200457.
